# SIRT1 Alleviates Oxidative Stress‐Induced Mitochondrial Dysfunction and Mitochondria‐Associated Membrane Dysregulation in Stress Urinary Incontinence

**DOI:** 10.1111/cpr.70009

**Published:** 2025-02-21

**Authors:** Liying Chen, Jianming Tang, Xiaohu Zuo, Bingshu Li, Cheng Liu, Shasha Hong, Jie Min, Ming Hu, Suting Li, Min Zhou, Mao Chen, Yong He, Ya Xiao, Xiaoyu Huang, Li Hong

**Affiliations:** ^1^ Department of Gynecology and Obstetrics Renmin Hospital of Wuhan University Wuhan People's Republic of China

**Keywords:** mitochondria‐associated membranes, mitochondrial biogenesis, mitophagy, SIRT1, stress urinary incontinence

## Abstract

The pathogenesis of stress urinary incontinence (SUI), a condition common in women, remains to be fully elucidated. This study revealed that the incidence of SUI is associated with mitochondrial homeostasis dysregulation following oxidative stress in the fibrous connective tissue of the pelvic floor. SIRT1 is an essential factor for maintaining mitochondrial homeostasis; however, its potential role and mechanism of action in SUI pathogenesis remain unclear. Both in vitro and in vivo, we observed that oxidative stress reduced SIRT1 expression to inhibit the PGC‐1α/NRF1/TFAM and PINK1/Parkin signalling pathways, eliciting impairment of mitochondrial biogenesis and mitophagy in L929 cells and SUI mice. Decreased SIRT1 levels induced endoplasmic reticulum (ER) stress and altered the structure of mitochondria‐associated membranes (MAMs), disrupting ER‐mitochondrial calcium homeostasis and exacerbting ROS accumulation. SIRT1 activation can restore mitochondrial function and the structure of MAMs and alleviate ER stress in fibroblasts, promoting anterior vaginal wall repair and improving urodynamic parameters in the SUI model. Our findings provide novel insights into the role and associated mechanism of SIRT1 in ameliorating oxidative stress‐induced mitochondrial dysfunction in fibroblasts of the anterior vaginal wall and propose SIRT1 as a potential therapeutic target for SUI.

## Graphical abstract

SIRT1 activates mitochondrial biogenesis and mitophagy by facilitating the PGC‐1α/NRF1/TFAM and PINK1/Parkin signalling pathways and maintains the structural stability of MAMs and the normal expression of calcium channel‐related proteins, which alleviate ER stress and restore mitochondrial function, ultimately improving urodynamic parameters in the SUI model.

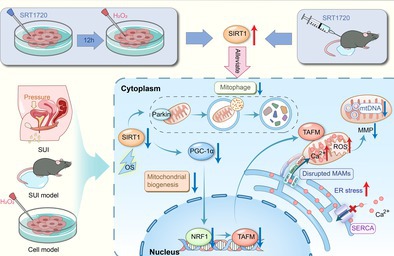

**Abbreviations**
BLPP: bladder leak point pressure
ECM: extracellular matrix
ER: endoplasmic reticulum
FUNDC1: FUN14 domain containing 1
GRP75: glucose regulated protein 75
GRP78: 78‐kDa glucose‐regulated protein
4‐HNE: 4‐hydroxynonenal
H₂O₂: hydrogen peroxide
IP3R: inositol 1,4,5‐triphate receptor
LC3B: microtubule associated protein 1 light chain 3 beta
MAMs: mitochondria‐associated membranes
MCU: mitochondrial calcium uniporter
mitoROS: mitochondrial reactive oxygen species
MMP: mitochondrial membrane potential
NRF1: nuclear respiratory factor 1
Nrf2: nuclear factor erythroid 2‐related factor 2
8‐OHdG: 8‐hydroxy‐2′‐deoxyguanosine
PERK: protein kinase R‐like endoplasmic reticulum kinase
PGC‐1α: peroxisome proliferator‐activated receptor gamma, coactivator 1 alpha
PINK1: PTEN‐induced putative kinase protein 1
POP: pelvic organ prolapse
ROS: reactive oxygen species
RT‐qPCR: reverse transcription‐quantitative real‐time PCR
SERCA2: sarcoplasmic/endoplasmic reticulum calcium ATPase 2
SIRT1: Sirtuin 1
SUI: stress urinary incontinence
TEM: transmission electron microscopy
TFAM: mitochondrial transcription factor 1
TUNEL: terminal deoxynucleotidyl transferase mediated dUTP nick end labeling
VD: vaginal dilatation
VDAC1: voltage‐dependent anion channel 1

## 1 Introduction

Stress urinary incontinence (SUI), the most common form of urinary incontinence in women, involves involuntary urine leakage during physical activities such as coughing, sneezing, laughing, or exercising, adversely impacting the quality of life of affected individuals and causing significant economic burdens [1, 2]. Recognised risk factors include childbirth, obesity, advanced age, diabetes, and smoking, although the exact pathophysiology and aetiology of SUI remain unclear [3, 4].

The normal function of the female pelvic organs relies on intact anatomical structures. Mechanical damage to the anterior vaginal wall disrupts the normal position and function of the bladder neck and urethra, leading to SUI and/or pelvic organ prolapse (POP) [5, 6, 7, 8, 9]. SUI and POP are associated with oxidative stress in pelvic supporting tissues, which may significantly alter extracellular matrix (ECM) metabolism in pelvic tissues [10, 11, 12]. Antioxidant therapy and Nrf2 overexpression have demonstrated promising therapeutic effects on ECM metabolism and cellular apoptosis in the anterior vaginal wall of a SUI mouse model [13]. Our validation of the pathological changes in the anterior vaginal wall of patients with SUI is consistent with previous findings in mouse SUI studies [13]. Therefore, we hypothesise that oxidative stress in the pelvic tissues is a critical pathological factor in SUI.

The accumulation of ROS generated during mitochondrial metabolism induces oxidative stress, affecting the electron transport chain and initiating mitochondria‐induced apoptosis, ultimately inducing cell death [14, 15, 16]. Enhancing mitophagy and promoting mitochondrial biogenesis are key for mitochondrial function [17]. Excessive ROS directly oxidises proteins within the endoplasmic reticulum (ER), inducing ER stress [18]. The ER and mitochondria structurally and functionally interact through mitochondria‐associated membranes (MAMs), crucial for calcium homeostasis [19]. The IP3R/GRP75/VDAC1 complex, a key structure located on MAMs, enhances its activity during ER stress, leading to excessive calcium transfer from the ER to the mitochondria, thereby triggering mitochondrial dysfunction [20, 21, 22]. However, whether regulation of mitochondrial function and alleviation of ER stress are involved in ECM metabolism in the pelvic tissues remains to be determined.

SIRT1 is a deacetylase that regulates oxidative stress and cellular senescence by maintaining mitochondrial function and mitigating ER stress [23, 24, 25, 26]. SIRT1 expression is reduced in uterosacral ligament fibroblasts from patients with POP, and SIRT1 activation reverses oxidative stress [27]. SIRT1 deacetylates and activates PGC‐1α, improving mitochondrial function and promoting energy production, thereby enhancing cellular resistance [28, 29]. SIRT1 inhibition increases mitochondrial protein acetylation, inhibits ubiquitin and LC3 recruitment, and suppresses mitophagy. MCU acetylation at K332 enhances the induction of mitochondrial calcium overload and cell death [24]. However, whether SIRT1 can mitigate SUI progression by maintaining the stability of the mitochondrial‐endoplasmic reticulum network remains to be determined.

In the present study, we investigated the role of SIRT1 in regulating mitochondrial function and MAMs in mitigating ER and oxidative stress both in vivo and in vitro. We postulate that SIRT1 upregulation is an important theoretical foundation for therapeutic studies on SUI. Herein, we identified attenuated collagen content, increased cellular apoptosis, and elevated oxidative damage in the anterior vaginal wall as key pathological characteristics of patients with SUI.

## 2 Materials and Methods

### 2.1 Patient Selection and Tissue Collection

The study received approval from the Ethics Committee of Renmin Hospital of Wuhan University (Wuhan, China), and informed consent was obtained from all participants before their involvement (WDRY2022‐K044). Twenty patients who underwent gynecological surgery in our hospital from March 2022 to March 2023 were selected, including 10 patients with SUI (no greater than stage II by POP‐Q, SUI group), and the remaining 10 patients underwent total hysterectomy for benign diseases (control group, without SUI or POP). During the surgical procedures, tissue specimens were obtained from the anterior vaginal wall and promptly sent to the laboratory within half an hour for subsequent experiments. Exclusion criteria for all participants included a history of endometriosis, gynecologic malignancies, pelvic inflammatory conditions, connective tissue disorders, emphysema, prior pelvic surgery, advanced pelvic organ prolapse (greater than stage II by POP‐Q), and application of oestrogen within the last 3 months. A full‐thickness tissue sample measuring 0.5 × 1.0 cm^2^ was excised from the anterior vaginal wall during surgery. A representative cross‐section of this tissue was fixed in 10% buffered formalin for 16 h, followed by paraffin embedding for immunohistochemistry. The remaining tissue was immediately frozen and stored in liquid nitrogen for further processing and experiments.

### 2.2 Animal Experimental Design

The study was conducted using female C57BL/6 mice, aged 10 weeks, which were selected as the subject population. All animal experiments and protocols were approved by the Institutional Animal Care and Ethics Committee of the Renmin Hospital of Wuhan University and conducted in accordance with the institutional guidelines of the Institutional Review Board (WDRM20200805). The mice were randomly assigned to one of four groups: an untreated control group without vaginal dilatation (VD) and SRT1720 administration (CON group), a group that underwent VD only (VD group), a group injected intraperitoneally with SRT1720 only (SRT1720 group), and a group that underwent VD followed by intraperitoneal injection with SRT1720 (VD+SRT1720 group). SRT1720 (Beyotime), dissolved in DMSO, was administered by intraperitoneal injection at 5 mg/kg for 7 days, starting from the 1st day after VD. Mice in the non‐SRT1720 treated groups received an equivalent dose of DMSO via the same method. As previously outlined in our research [30], the VD method was employed to create a SUI mice model for subsequent experimentation. On the seventh day following VD, bladder leak point pressure (BLPP) was measured to ascertain the efficacy of the SUI model and the therapeutic effect of SRT1720. Finally, the anterior vaginal wall tissues were harvested post‐sacrifice for further studies.

### 2.3 Cell Culture and Treatments

L929 cells, which are fibroblasts derived from the connective tissue of mice, were obtained from China Center for Type Culture Collection (Wuhan) and cultured in MEM (Wuhan Pricella Biotechnology Co. Ltd.), with the addition of 10% fetal bovine serum (FBS, Gemini Bio‐Products, CA, USA) and 1% double antibiotics (100 KU/ml penicillin G and 100 mg/mL streptomycin; Genom Biotech Ltd.). The culture conditions were maintained at 37°C with 5% CO_2_, and the medium was refreshed every 2 days. The cells were passaged or cryopreserved once they reached approximately 70%. Cells in the exponential growth phase were exposed to different concentrations of H_2_O_2_ (Sigma‐Aldrich CO., St Louis, MO, USA) for 4 h. In addition, cells were exposed to 2 μM SRT1720 (Beyotime) for 12 h before receiving H_2_O_2_ treatment.

### 2.4 siRNA Transfection

L929 cells were seeded in 6‐well plates and subsequently transfected with small interfering RNAs (siRNAs). According to the manufacturer's protocol, the siRNAs (40 nM) were transfected into L929s using InvitroRNA. The siRNA sequences were designed and synthesised by Genepharma Co. Ltd. (Suzhou, China). The forward (F) primer sequence of SIRT1 siRNA is GCACCGAUCCUCGAACAAUTT, and the reverse (R) primer sequence is AUUGUUCGAGGAUCGGUGCTT.

### 2.5 Western Blot Analysis

The total protein from L929 cells and anterior vaginal wall tissues was isolated using RIPA Lysis Buffer (Servicebio, Wuhan, China) with PMSF (Servicebio, Wuhan, China). The concentration of the extracted protein was subsequently determined using the BCA protein assay kit (Beyotime Institute of Biotechnology, Haimen, China). For analysis, protein samples (20 μg) were separated by SDS‐PAGE (10%) and then transferred onto a PVDF membrane (0.45 μm, Pall, USA). After being blocked with 5% skim milk for 1 h, the membranes were incubated overnight at 4°C with primary antibodies against SIRT1 (1:1000, Proteintech Group Inc.), PGC‐1α (1:1000, Proteintech Group Inc.), NRF1 (1:5000 Proteintech Group Inc.), TFAM (1:5000, Proteintech Group Inc.), PINK1 (1:2000, Proteintech Group Inc.), Parkin (1:2000, Proteintech Group Inc.), Collagen I (1:1000, Proteintech Group Inc.), Collagen III (1:1000, Proteintech Group Inc.), Cytochrome c (1:1000, Proteintech Group Inc.), VDAC1 (1:2000, Proteintech Group Inc.), GRP75 (1:5000, Proteintech Group Inc.), FUNDC1 (1:1000, Cell Signalling Technology Inc.), PERK (1:1000, Proteintech Group Inc.), GRP78 (1:3000, Proteintech Group Inc.), SERCA2 (1:5000, Proteintech Group Inc.), MCU (1:3000, Proteintech Group Inc.) and β‐actin (1:20,000, Proteintech). Subsequently, the membranes were incubated with HRP‐conjugated goat anti‐mouse IgG or goat anti‐rabbit IgG for 1 h at room temperature. Finally, the immunoreactive bands were treated with an enhanced chemiluminescent substrate (Pierce Fast Western Blot Kit, catalog no. 35055, Thermo Scientific, Waltham, MA, USA) and detected with the ChemiDoc Imaging System (Bio‐Rad Laboratories Inc., CA, USA).

### 2.6 Reverse Transcription‐Quantitative Real‐Time PCR (RT‐qPCR)

Total RNA was extracted from L929 cells and anterior vaginal wall tissues using the TRIzol reagent (Invitrogen; Thermo Fisher Scientific Inc., Waltham, MA, USA) following the manufacturer's instructions. The extracted RNA samples were reversely transcribed into complementary cDNA using the Hifair III 1st Strand cDNA Synthesis SuperMix for qPCR (gDNA digester plus) (Yeasen Biotechnology (Shanghai) Co. Ltd.). The primers used in the study were synthesised and obtained from Sangon Biotech (Shanghai) Co. Ltd., with their sequences detailed in Table S1. Gene expression levels were then assessed through qRT‐PCR using the Hifair qPCR SYBR Green Master Mix (No Rox) (Yeasen Biotechnology (Shanghai) Co. Ltd.) and an Applied Biosystems 7500 Real‐Time system (Applied Biosystems, Thermo Fisher Scientific Inc.). The relative mRNA levels were quantified by the 2^−△△Ct^ method, with GAPDH serving as the internal control.

### 2.7 Flow Cytometry

To investigate mitochondrial reactive oxygen species (mitoROS), cells were treated, harvested, and stained with MitoSOX Red agent (5 μM, Invitrogen Corp.) for 10 min at 37°C. Following staining, the cells underwent two washes with PBS, and then they were resuspended in PBS and analysed by a CytoFLEX flow cytometer (Beckman Coulter, USA). The excitation and emission wavelengths were set as 510/580 nm. The relative mean fluorescence intensities were then processed and analysed using FlowJo software, version 10 (TreeStar Inc., Ashland, OR, USA).

To quantify the mitochondrial membrane potential (MMP) in L929 cells, the JC‐1 assay (Beyotime Biotechnology) was employed. The cells were harvested from the culture plate after treatment. After a 30‐min incubation period at 37°C, the monomeric green fluorescence emissions and aggregate red fluorescence intensities in cells were monitored at Ex/Em = 490/530 and 525/590 nm by a CytoFLEX flow cytometer (Beckman Coulter, USA). The MMP for each group was calculated as the ratio of red to green fluorescence and expressed relative to the control group.

The overall reactive ROS levels in L929 cells were assessed using a Reactive Oxygen Species Assay Kit (Beyotime Biotechnology). After treatment, cells were harvested and incubated with 2,7‐dichlorodi‐hydrofluorescein diacetate (DCFH‐DA) for 20 min at 37°C. The cells were washed three times with an FBS‐free medium and then resuspended in flow buffer. The fluorescence intensity in the cells was monitored by a CytoFLEX flow cytometer (Beckman Coulter, USA) with excitation and emission wavelengths of 488 and 525 nm, respectively. The relative mean fluorescence intensities were subsequently analysed with FlowJo software, version 10 (TreeStar Inc., Ashland, OR, USA).

An apoptosis assay was performed using the Annexin V‐PE/7‐AAD Apoptosis Detection Kit (Yeasen Biotechnology (Shanghai) Co. Ltd.). Cells were seeded into 6‐well plates at a density of 1.5 × 10^6^ cells per well. Subsequent to the designated treatment, all cells in both the supernatant and adherent populations were collected, washed three times with PBS, and resuspended in 1× binding buffer. An additional 5 μL of Annexin V/PE and 10 μL of 7‐amino‐actinomycin (7‐AAD) were then added to the tube. Subsequently, the cells were gently vortexed and incubated for 15 min at room temperature in the dark. Finally, 400 μL of 1× binding buffer was added, mixed thoroughly, and the samples were analysed using the CytoFLEX flow cytometer (Beckman Coulter, USA). Data analysis was conducted using FlowJo software, version 10 (TreeStar Inc., Ashland, OR, USA).

### 2.8 Mitochondrial DNA (mtDNA) Content Measurement

The total DNA from specimens and cell lysis was isolated using the MolPure Cell/Tissue DNA Kit (Yeasen Biotechnology (Shanghai) Co. Ltd.). The expression levels of the mitochondrial genes (mtAtp6 in mice and ND4 in humans) and nuclear genes (Tert in mice and GAPDH in humans) were quantified through RT‐qPCR in cells and tissues. Primer sequences, which were synthesised by Sangon Biotech (Shanghai) Co. Ltd., are detailed in Table S2. The relative mitochondrial content was determined by calculating the ratio of mitochondrial DNA expression to nuclear DNA expression.

### 2.9 Cell Viability Assay

Cell viability was assessed utilising the SuperKine Maximum Sensitivity Cell Counting Kit‐8 (CCK‐8, Abbkine Scientific Co. Ltd). At the conclusion of the exposure period, a mixture comprising 100 μL of medium and 10 μL of CCK‐8 solution was added to each well of the 96‐well culture plates. Subsequently, the cells were incubated at 37°C for 1–2 h. Absorbance at 450 nm was measured using the EnSight multimode plate reader (PerkinElmer, USA). The results were expressed as a percentage relative to the absorbance of the untreated control group.

### 2.10 Masson's Trichrome Staining

The anterior vaginal walls were embedded in paraffin and subsequently sectioned into 4‐μm transverse slices. These sections were then subjected to Masson's trichrome staining (Masson Stain Kit HT15, Sigma, USA), following the standard protocol outlined in the manufacturer's instructions. This staining technique renders collagen fibres bright green, facilitating their identification on histological slides. To quantitatively assess the collagen fibre content, the mean optical density (MOD) of the positively stained areas was measured using ImageJ software (NIH, Bethesda, MD, USA).

### 2.11 Transmission Electron Microscopy (TEM) Examination

Anterior vaginal wall samples were initially cut into small pieces (0.8–1.0 mm^3^) and fixed in 2.5% pre‐cooled glutaraldehyde for a duration of 6 h at a temperature of 4°C. Following fixation, the samples underwent a dehydration process using graded ethanol and acetone. They were then infiltrated with a mixture of half propylene oxide and embedded in resin. Sections with a thickness of 50 nm were cut and collected on grids for further analysis. Finally, the grids were stained with 4% uranyl acetate for 15–30 min and 0.5% ad citrate for 3–15 min. The ultrastructural feature of the anterior vaginal wall was subsequently examined using transmission electron microscopy (Hitachi Ltd.). Quantitative analysis of MAM coverage and distance per cell area was conducted using ImageJ as described [31].

### 2.12 Immunohistochemical (IHC) Staining

The anterior vaginal wall tissues were deparaffinised, rehydrated, and immersed in sequence. The sections were then incubated in a 10% goat serum sealant to block non‐specific binding sites. Primary antibodies were applied, including anti‐SIRT1 (1:200), anti‐PGC‐1α (1:150), anti‐NRF1 (1:50), anti‐TFAM (1:200), anti‐4HNE (1:200) and anti‐8‐OHDG (1:200), and the sections were incubated overnight at 4°C. The sections were then incubated with biotin‐labelled goat anti‐rabbit/mouse IgG working solutions (1:500) for 2 h at room temperature. The slides were incubated with Streptavidin Biotin HRP Complex and then stained at room temperature using pre‐concentrated DAB chromogenic solutions. Finally, tissues were visualised using Upright Microscopes (BX53, Olympus), and protein expression levels were quantified with Image J software.

### 2.13 TUNEL Assay

The evaluation of apoptotic cells in the tissue of the anterior vaginal wall was evaluated using terminal deoxynucleotidyl transferase (TdT)‐mediated dUTP‐biotin nick end labeling (TUNEL) staining with an In Situ Cell Death Detection Kit (Roche, Mannheim, Germany), in accordance with the manufacturer's instructions. Apoptotic cells were identified by the presence of brown granules within the nuclei, with or without concomitant slight cytoplasmic staining. ImageJ software was used for the analysis and quantification of apoptotic cell rates in vaginal wall tissues. The apoptotic cell rate was calculated as the percentage of apoptotic cells relative to the total number of cells.

### 2.14 Immunofluorescence Staining

L929 cells were inoculated into 6‐well plates and treated with H₂O₂, either in the presence or absence of SRT1720. After a 10‐min fixation with 4% formaldehyde, the cells underwent permeabilization using 0.3% Triton X‐100 for an additional 10 min, followed by blocking with 5% BSA for 30 min at room temperature. The anterior vaginal wall tissues were collected from each group after perfusion with ice‐cold PBS 1 day after 7 days of VD. These tissues were then fixed with 4% paraformaldehyde for 48 h and subsequently cut into slices. The slices were incubated in 0.3% Triton X‐100 for 10 min and blocked with 5% fetal bovine serum for 60 min at room temperature. Then, cells were incubated with anti‐SIRT1 (1:100), anti‐PGC‐1α (1:100), anti‐NRF1 (1:100), anti‐TFAM (1:100), anti‐collagen I (1:100) and anti‐collagen III (1:100), anti‐PINK1 (1:100), anti‐parkin (1:100), anti‐IP3R (1:100) and anti‐VDAV1 (1:100) overnight at 4°C. The slides were incubated with anti‐PINK1 (1:100) and anti‐parkin (1:100) overnight at 4°C. Next, cells and slides were further incubated with Cy3‐labelled or FITC‐labelled goat anti‐rabbit or anti‐mouse IgG (1:200, Wuhan Servicebio Technology Co. Ltd) for 1 h in the dark. Mitochondria and nuclei were labelled with Mito‐tracker and DAPI, respectively. The images were examined using an upright microscope (BX53, Olympus) and a confocal laser scanning microscope (FV1200, Olympus). Data analysis was conducted using ImageJ software.

### 2.15 The Staining of Ca^2+^ in Mitochondria

To label the mitochondrial Ca^2+^ in L929 cells, Rhod‐2, AM Cell Permeant (Yeasen Biotechnology (Shanghai) Co. Ltd.) was utilised. Cells were cultured in confocal Petri dishes. The medium was removed, and the cells were washed three times with buffer solution. The Rhod‐2/AM working solution (2 μM) was added, and the cells were incubated at 37°C for 30 min. Following the incubation, the Rhod‐2/AM solution was removed, and the cells underwent three additional washes with the buffer solution. The buffer was added to cover the cells, and the incubation process was initiated at 37°C for 20–30 min. Fluorescence detection was performed using a confocal laser scanning microscope (FV1200, Olympus) and the data were analysed with Image J software.

For quantifying the mitochondrial Ca^2+^ concentration in L929 cells, the GENMED Intracellular Calcium Ion Concentration in Mitochondria Fluorescence Quantitative Assay Kit (product no. GMS10153.1) was employed, adhering to the manufacturer's instructions. The cells were seeded into black 96‐well plates, which were categorised into three groups: cell sample wells, blank control wells (devoid of cells), and maximum control wells (containing cells). Subsequently, Reagent A, D, E and the GENMED staining working solution were added to the corresponding wells followed by a 30‐min incubation at 37°C in the dark. Reagent A was then added to the cell sample wells, and the process was repeated. The samples were incubated at 37°C for 30 min. Reagent F was then added to the maximal control wells and incubated at 37°C for 15 min. The black 96‐well plate was then placed in a fluorometer (excitation wavelength 550 nm, emission wavelength 590 nm) to measure the relative fluorescence unit (RFU), which was then used to calculate the concentration of mitochondrial Ca^2+^ in L929 cells.

### 2.16 Statistical Analysis

The results are presented as the mean ± standard deviation (SD) derived from at least three independent experiments. Statistical analyses were conducted using one‐way analysis of variance (ANOVA) and visualised with Graph Pad Prism 9.0 software (GraphPad Software Inc., San Diego, CA, USA). When ANOVA indicated statistically significant differences, Dunnett's test was conducted to facilitate a comparison of the mean values between each of the two study groups. The levels of mitochondria‐associated signalling molecules in the anterior vaginal walls were compared using an independent samples Student's *t*‐test. A *p* value of less than 0.05 was considered to indicate statistically significant.

## 3 Results

### 3.1 Decreased Collagen Content, Increased Cellular Apoptosis, and Elevated Oxidative Damage and ER Stress in the Anterior Vaginal Wall Are Key Pathological Characteristics of Patients With SUI

**FIGURE 1:**
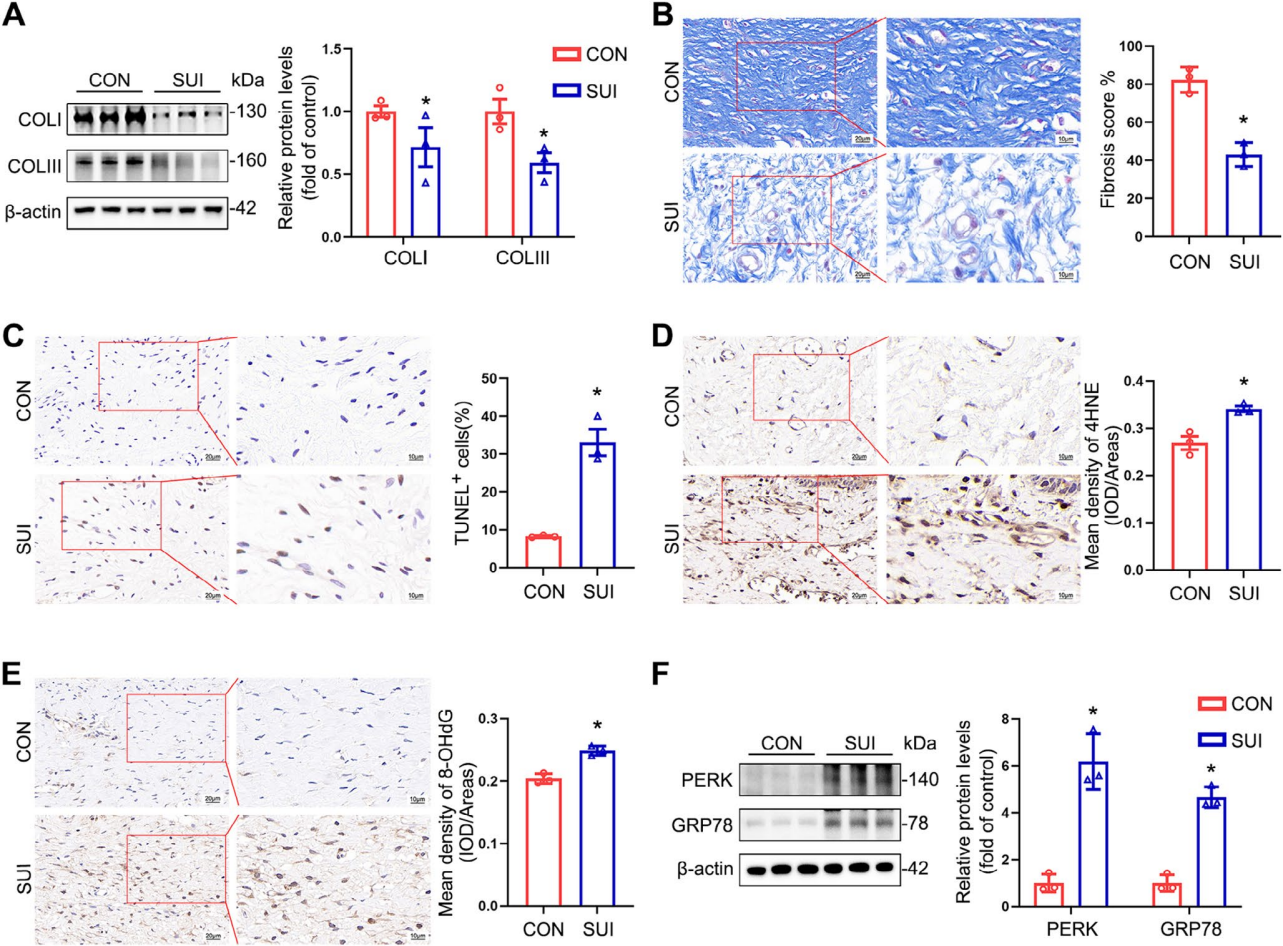
Pathological characteristics of the anterior vaginal wall in patients with SUI. (A) Immunoblot analysis of collagen I (COL I) and collagen III (COL III) in the anterior vaginal wall tissues. Quantification represents the levels of the indicated protein normalised to β‐Actin. (B) Masson staining of collagen fibres in the anterior vaginal wall tissues of both the control and SUI groups. (C) Apoptosis detection in the anterior vaginal wall tissues of both groups was performed using a TUNEL assay kit. (D, E) Representative images and quantification of IHC staining for 4‐HNE (D) and 8‐OHdG (E) in anterior vaginal wall tissues. (F) Immunoblot analysis of PERK and GRP78 in the anterior vaginal wall tissues. Quantification represents the levels of the indicated protein normalised to β‐Actin. CON: Control group; SUI: Sui group. Data are expressed as mean ± SD. *p < 0.05 compared with the control group.

We observed a significant downregulation of collagens I and III in the anterior vaginal wall of patients with SUI (Figure 1A); the collagen fibres were broken, discontinuous, and loosely arranged in a disordered manner (Figure 1B). Next, we determined the cellular apoptotic rates in the anterior vaginal wall of patients with SUI and observed markedly upregulated rates (Figures 1C and S1A). In addition, we analysed the levels of oxidative damage markers in the same patients and found that 4‐HNE, a lipid oxidative damage marker, and 8‐OHdG, a DNA oxidative damage marker, were significantly upregulated compared with controls (Figure 1D,E). We compared the expression of ER stress indicators, PERK and GRP78, in the anterior vaginal wall tissue of patients with SUI and those without. Our findings revealed higher levels of ER stress in the anterior vaginal wall of patients with SUI (Figure 1F). Therefore, we speculate that oxidative damage is associated with the induction of cellular apoptosis and ER stress, which deteriorate the anterior vaginal wall in SUI, and that these indices are crucial for the pathogenesis of SUI.

### 3.2 SIRT1 Downregulation and Mitochondrial Dysfunction in the Anterior Vaginal Wall of Patients With SUI

**FIGURE 2:**
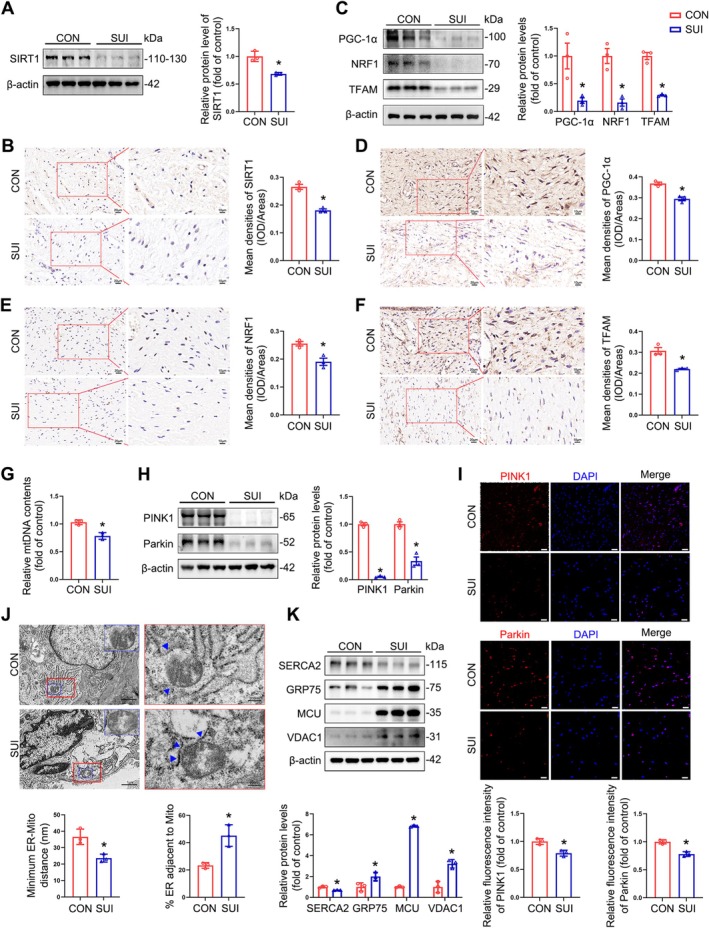
Downregulation of SIRT1 and mitochondrial dysfunction in the anterior vaginal walls of patients with SUI. (A) Immunoblot analysis of SIRT1 in anterior vaginal wall tissues in patients with/without SUI. Quantification represents the level of the indicated protein normalised to β‐Actin. (B) Representative images of the IHC staining for SIRT1 in anterior vaginal wall tissues were obtained and quantified. (C) Immunoblot analyses of PGC‐1α, NRF1 and TFAM in anterior vaginal wall tissues in patients with/without SUI. Quantification represents the levels of the indicated protein normalised to β‐Actin. (D–F) Representative images of the IHC staining for PGC‐1α, NRF1 and TFAM from the anterior vaginal wall tissues. (G) RT‐qPCR analyses of relative mtDNA contents in anterior vaginal wall tissues in patients with/without SUI. (H, I) Immunoblot (H) and immunofluorescence (I) analyses of PINK1/Parkin in anterior vaginal wall tissues in patients with/without SUI. Scale bar = 20 μm. (J) Representative TEM images of mitochondrial ultrastructure in fibroblasts from the anterior vaginal wall. MAMs were indicated by blue arrows. Quantitative analysis of MAM parameters, including MAM distance and length to mitochondrion was performed on the TEM images. (K) Immunoblot analyses of calcium channel SERCA2, GRP75, VDAC1 and MCU in anterior vaginal wall tissues in patients with/without SUI. Quantification represents the levels of the indicated protein normalised to β‐Actin. CON: Control group; SUI: SUI group. Data are expressed as mean ± SD. *p < 0.05 compared with control group.

Oxidative stress is closely correlated with mitochondrial dysfunction, and SIRT1 is an important regulator of mitochondrial function [32]. To evaluate the role of SIRT1 in the anterior vaginal wall of patients with SUI, we determined the total mRNA and protein levels of SIRT1. The expression of SIRT1 was significantly reduced in tissues from women with SUI (Figures S1B and 2A); immunohistochemistry revealed consistent results (Figure 2B). Previous studies have shown that SIRT1 interacts with PGC‐1α and stimulates mitochondrial biogenesis, thereby maintaining mitochondrial function through the PGC‐1α/NRF1/TFAM signalling pathway [33]. We, therefore, examined the expression of PGC‐1α, NRF1, and TFAM in the anterior vaginal wall of patients with SUI and found that mRNA and protein levels were attenuated compared with those in control patients (Figures S1C and 2C); our immunohistochemical data also supported the above results (Figure 2D–F). Emerging evidence suggests that mitochondrial biogenesis is closely associated with mtDNA content [33]. Our RT‐qPCR results revealed a reduction in the relative amount of mtDNA in the anterior vaginal wall of patients with SUI (Figure 2G). In addition, we analysed the levels of proteins associated with mitophagy in the anterior vaginal wall of patients with SUI and observed that the expression of PINK1 and Parkin was significantly reduced compared with the control group, and immunofluorescence showed congruent results (Figure 2H,I). Notably, TEM revealed a significant increase in fragmented and vacuolated mitochondria, the diminished distance between the mitochondria and the ER, and the augmented length of MAMs in the tissues of the anterior vaginal wall in the SUI group compared with the control group (Figure 2J). In addition, we investigated the expression levels of calcium channels in the ER membrane (SERCA2), mitochondria (VDAC1 and MCU), and ER‐mitochondrial Ca^2+^ transfer factors (GRP75). Our findings revealed elevated levels of GRP75, MCU, and VDAC1 and decreased level of SERCA2 in the anterior vaginal wall of patients with SUI compared with the control group, suggesting disturbed calcium homeostasis (Figure 2K). These findings indicate that a reduction in SIRT1 expression in fibroblasts of the anterior vaginal wall of patients with SUI may cause mitochondrial dysfunction and disturbed calcium homeostasis.

### 3.3 Oxidative Stress Induces Abnormalities in Mitochondrial Morphology and Mitochondrial Dysfunction by Inhibiting the Expression of SIRT1

**FIGURE 3:**
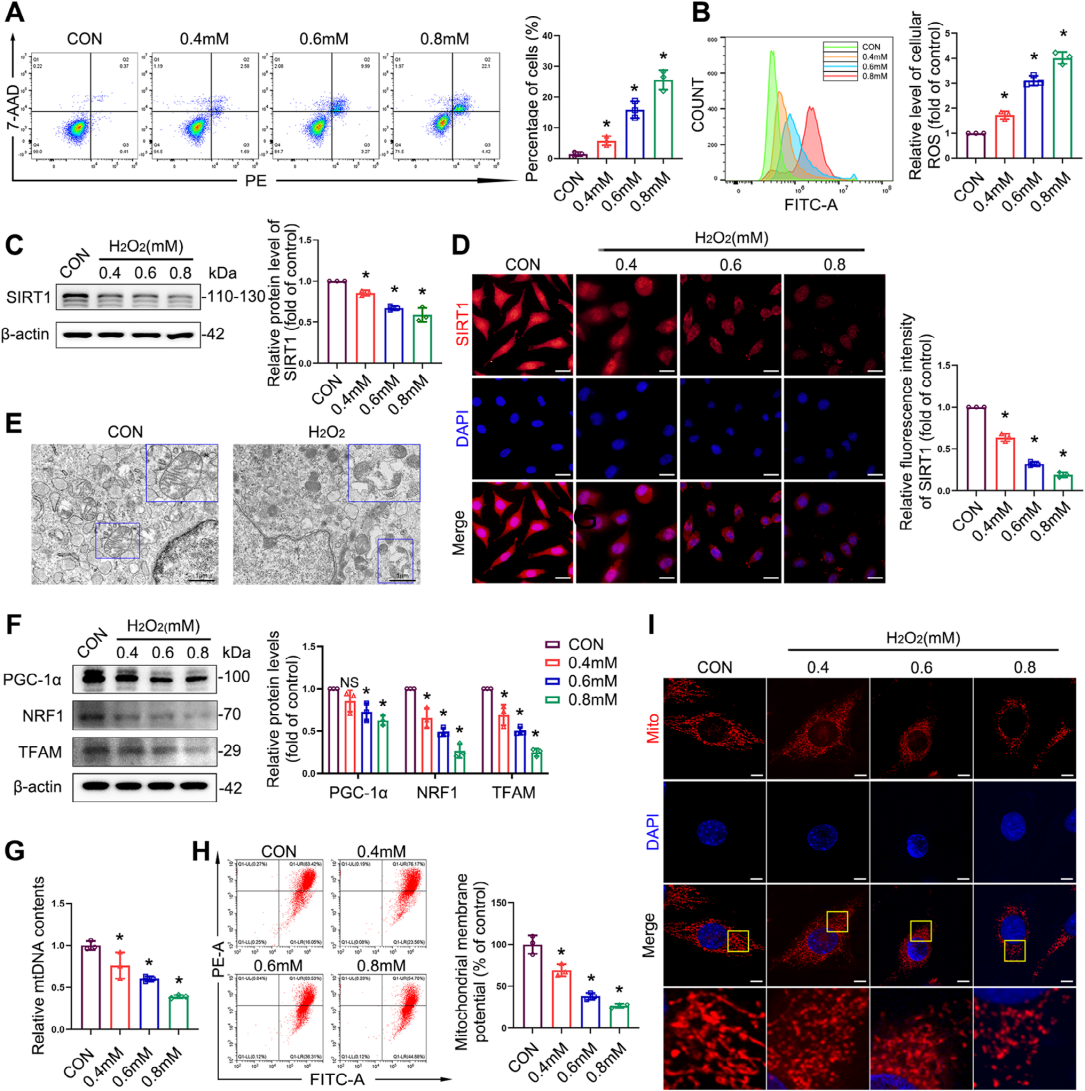
Oxidative stress inhibits SIRT1 expression and induces abnormalities in mitochondrial morphology and mitochondrial biogenesis. L929 cells were treated with 0.4, 0.6, and 0.8 mM H_2_O_2_ for 4 h. (A) Flow cytometry analysis of cellular apoptotic rates. (B) Representative flow plots of ROS production in L929 cells measured by flow cytometry. (C) Immunoblot analysis of SIRT1 in L929 cells. Quantification represents the level of the indicated protein normalised to β‐Actin. (D) Immunofluorescence staining of SIRT1 in L929 cells. Scale bar = 20 μm. (E) Representative images of mitochondrial ultrastructure in L929 cells treated with or without H_2_O_2_ (0.6 mM), shown by TEM. (F) Immunoblot analyses of PGC‐1α, NRF1, and TFAM in L929 cells. Quantification represents the levels of the indicated proteins normalised to β‐Actin. (G) RT‐qPCR analyses of relative mtDNA contents in L929 cells. (H) Representative flow plots of MMP levels in L929 cells measured by flow cytometry. (I) Representative images from MitoTracker‐stained control and H_2_O_2_‐treated L929 cells. Scale bars = 5 μm. Data are expressed as mean ± SD. *p < 0.05 compared with the control group. NS: No significant difference was observed.

To determine the effects of oxidative stress on SIRT1 expression in fibroblasts, we established a cellular model of oxidative stress in L929 cells using hydrogen peroxide (H_2_O_2_). H_2_O_2_ significantly reduced the activity of L929 cells in a concentration‐ and time‐dependent manner (Figure S2A). There was also a significant increase in apoptosis commensurate with increasing H_2_O_2_ concentrations (Figure 3A). The levels of intracellular ROS increased as the concentration of H_2_O_2_ increased, suggesting that oxidative damage was induced by H_2_O_2_ treatment (Figure 3B). Next, we measured SIRT1 mRNA and protein levels in L929 cells (Figures S2B and 3C) and found that both were significantly downregulated in H_2_O_2_ groups compared with control cells. SIRT1 immunofluorescence was similarly significantly reduced in H_2_O_2_ groups (Figure 3D). Furthermore, oxidative stress induced ultrastructural damage to the mitochondria, including mitochondrial swelling and loss of cristae (Figure 3E). To determine whether mitochondrial functions, such as mitochondrial biogenesis and mitophagy, were impaired by oxidative stress, we assessed the related signalling pathways in L929 cells. Our results indicated that mRNA and protein levels were reduced in the H_2_O_2_ groups compared with the control group (Figures S2C and 3F). We obtained consistent results from the immunofluorescence assay (Figure S2D–F). RT‐qPCR revealed a reduction in the relative amount of mtDNA in the H_2_O_2_ groups (Figure 3G). MMP was reduced in the H_2_O_2_ groups compared with the control group (Figure 3H). Treatment with H_2_O_2_ reduced the number of mitochondria and fragmented the mitochondria (Figures 3I and S2G).

**FIGURE 4:**
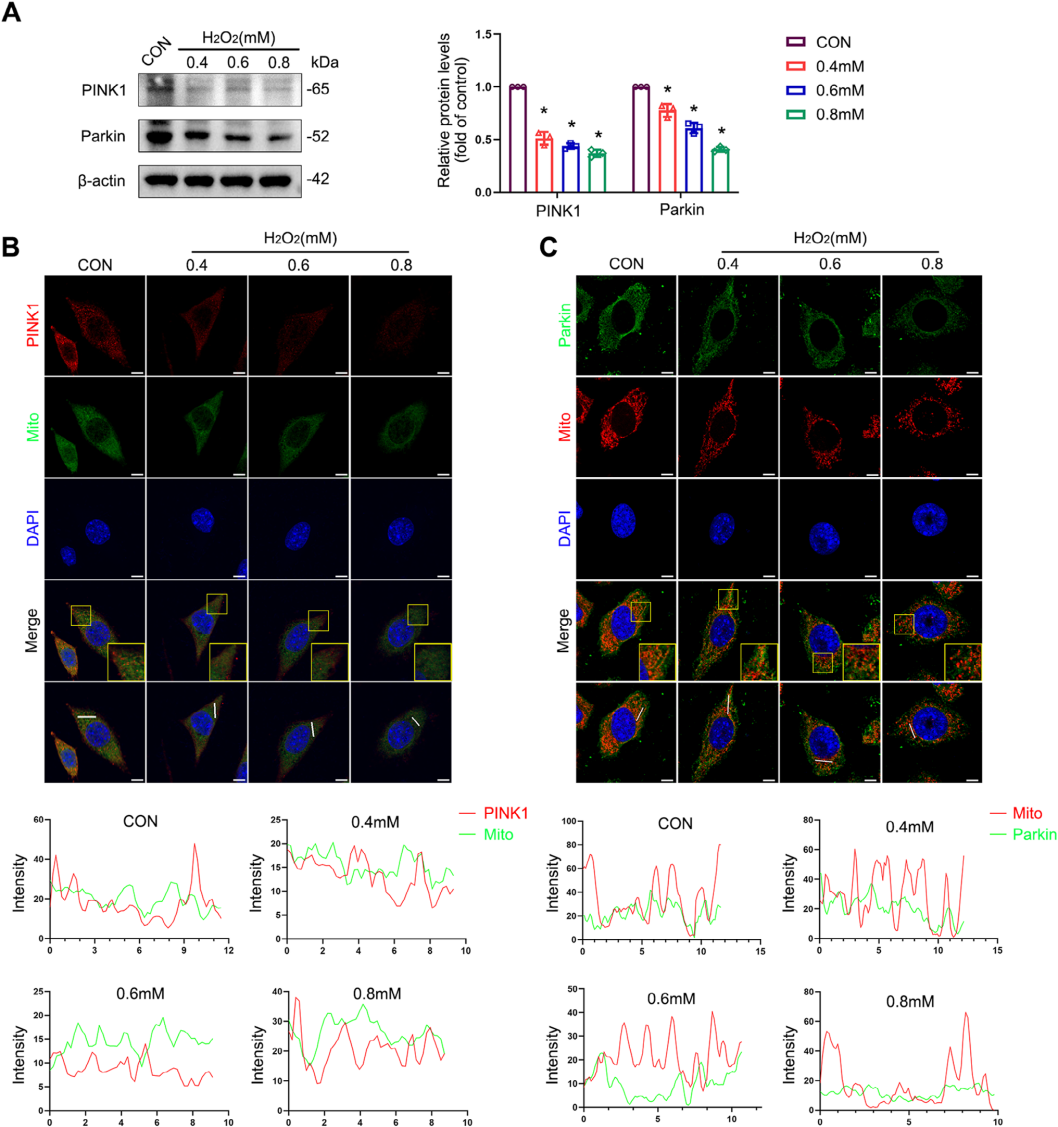
Oxidative stress‐induced SIRT1 inhibition impairs mitophagy. (A) Immunoblot analyses of PINK1 and Parkin in L929 cells. Quantification represents the levels of the indicated proteins normalised to β‐Actin. (B, C) Co‐localization analysis of immunofluorescence images of PINK1 (red) and MitoTracker (green) (B), Parkin (green) and MitoTracker (red) (C) in different groups of L929 cells after being treated with H_2_O_2_ for 4 h. Data are expressed as mean ± SD. *p < 0.05 compared with control group.

To verify whether mitophagy was impaired by oxidative stress, we examined the protein levels of PINK1 and Parkin following H_2_O_2_ treatments and found that they were significantly diminished (Figure 4A), and immunofluorescence also showed similar results (Figure S2H). When mitophagy is initiated, PINK1 accumulates on the outer membranes of dysfunctional mitochondria. However, in this study, the mitochondrial recruitment of PINK1 was inhibited with increasing H_2_O_2_ concentrations (Figure 4B). Subsequently, PINK1 induced Parkin translocation from the cytoplasm to the mitochondria; however, mitochondrial Parkin translocation was inhibited by H_2_O_2_ (Figure 4C).

Collectively, these findings suggest that oxidative stress induces abnormalities in mitochondrial morphology and function by inhibiting the expression of SIRT1.

### 3.4 Oxidative Stress Induces ER Stress and Disrupts the Structure of MAMs, Leading to Mitochondrial Calcium Overload and Exacerbated Mitochondrial ROS Accumulation

**FIGURE 5:**
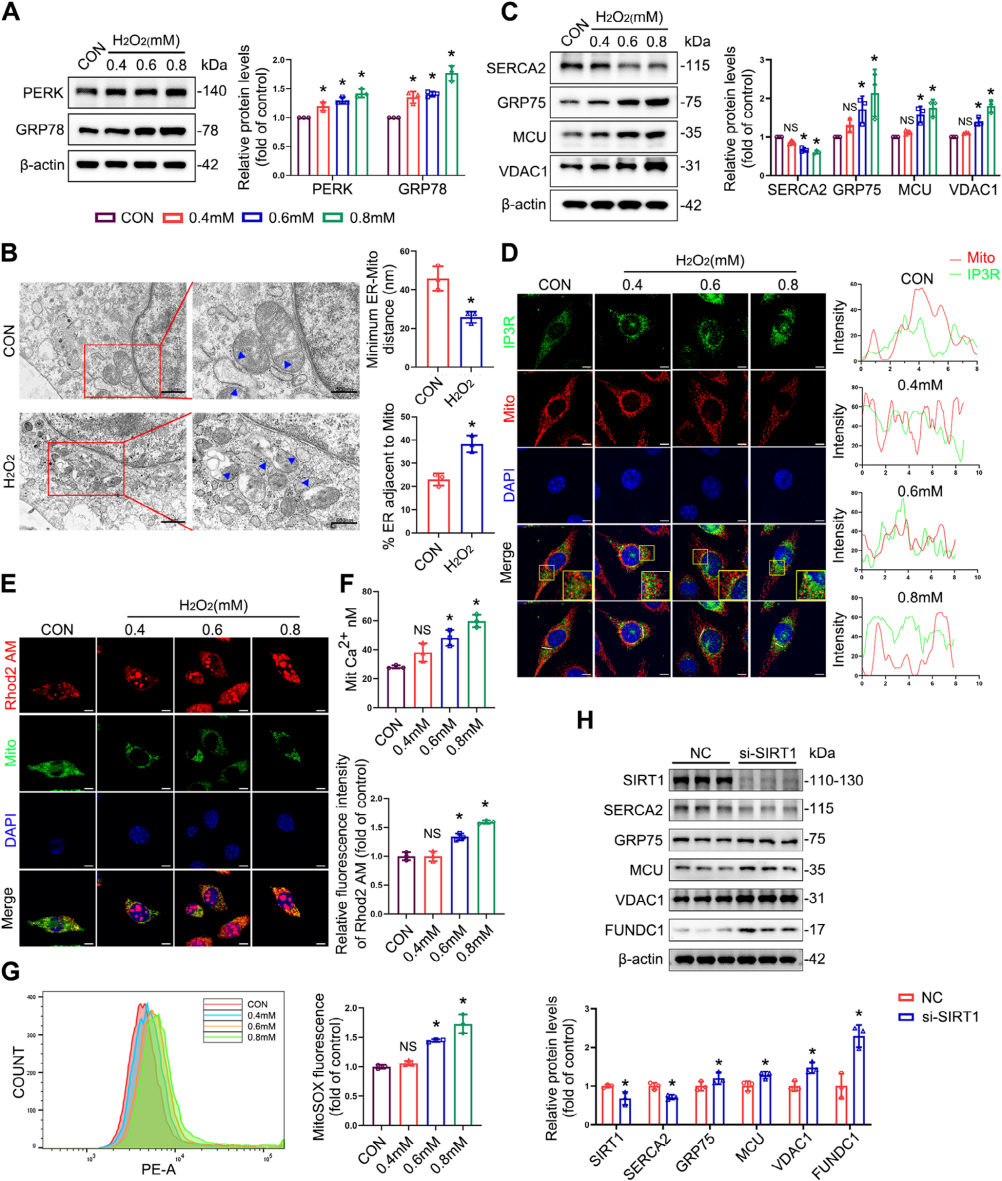
The structural and functional alterations of MAMs and calcium homeostasis in L929 cells following oxidative stress. (A) Immunoblot analyses of PERK and GRP78 in L929 cells. Quantification represents the levels of the indicated proteins normalised to β‐Actin. (B) Representative TEM images of mitochondrial ultrastructure in L929 cells. MAMs were indicated by blue arrows. Quantitative analysis of MAM parameters, including MAM distance and length to mitochondrion in TEM images. (C) Immunoblot analyses of SERCA2, GRP75, MCU, and VDAC1 in L929 cells. Quantification represents the levels of the indicated proteins normalised to β‐Actin. (D) Co‐localization analysis of immunofluorescence images of IP3R (green) and MitoTracker (red) in different groups of L929 cells following treatment with H_2_O_2_ for 4 h. (E) Co‐localization fluorescence imaging was conducted with MitoTracker, demonstrating that Rhod‐2 AM is predominantly an indicator of mitochondrial Ca^2+^ levels. (F) Concentration of mitochondrial calcium ions. (G) Representative flow plots of mitochondrial ROS production in L929 cells measured by flow cytometry. (H) Immunoblot analyses of SIRT1, SERCA2, GRP75, MCU, VDAC1 and FUNDC1 expression in L929 cells after SIRT1 knockdown. Quantification represents the levels of the indicated proteins normalised to β‐Actin. Data are expressed as mean ± SD. *p < 0.05 compared with control group. NS: No significant difference was observed.

L929 cells exhibited enhanced ER stress following treatment with H_2_O_2_ (Figure 5A). Besides, TEM showed that the distance between the mitochondria and the ER was diminished, while the length of MAMs was augmented in the presence of H₂O₂ (Figure 5B). H_2_O_2_ treatment increased the expression levels of VDAC1, MCU and GRP75 and downregulated SERCA2 (Figure 5C). In addition, we observed enhanced co‐localization of the ER protein IP3R with VDAC1 and mitochondria in H_2_O_2_ groups (Figures 5D and S2I). H₂O₂ treatment induced mitochondrial calcium overload (Figure 5E,F) and increased mitochondrial ROS generation (Figure 5G). SIRT1 knockdown in L929 cells resulted in a significantly increased expression of MAM‐tethering proteins and mitochondrial ER calcium channel‐related proteins and a decrease in SERCA2 (Figure 5H), indicating a crucial role for SIRT1 in the structural integrity of MAMs and calcium homeostasis under oxidative stress. This may be linked to impaired calcium transfer due to ER stress and alterations in the structure of MAMs, indicating a potential connection to SIRT1 inhibition.

### 3.5 SIRT1 Upregulation Alleviates Abnormalities in Mitochondrial Morphology and Reduces Mitochondrial Dysfunction Caused by Oxidative Stress

**FIGURE 6:**
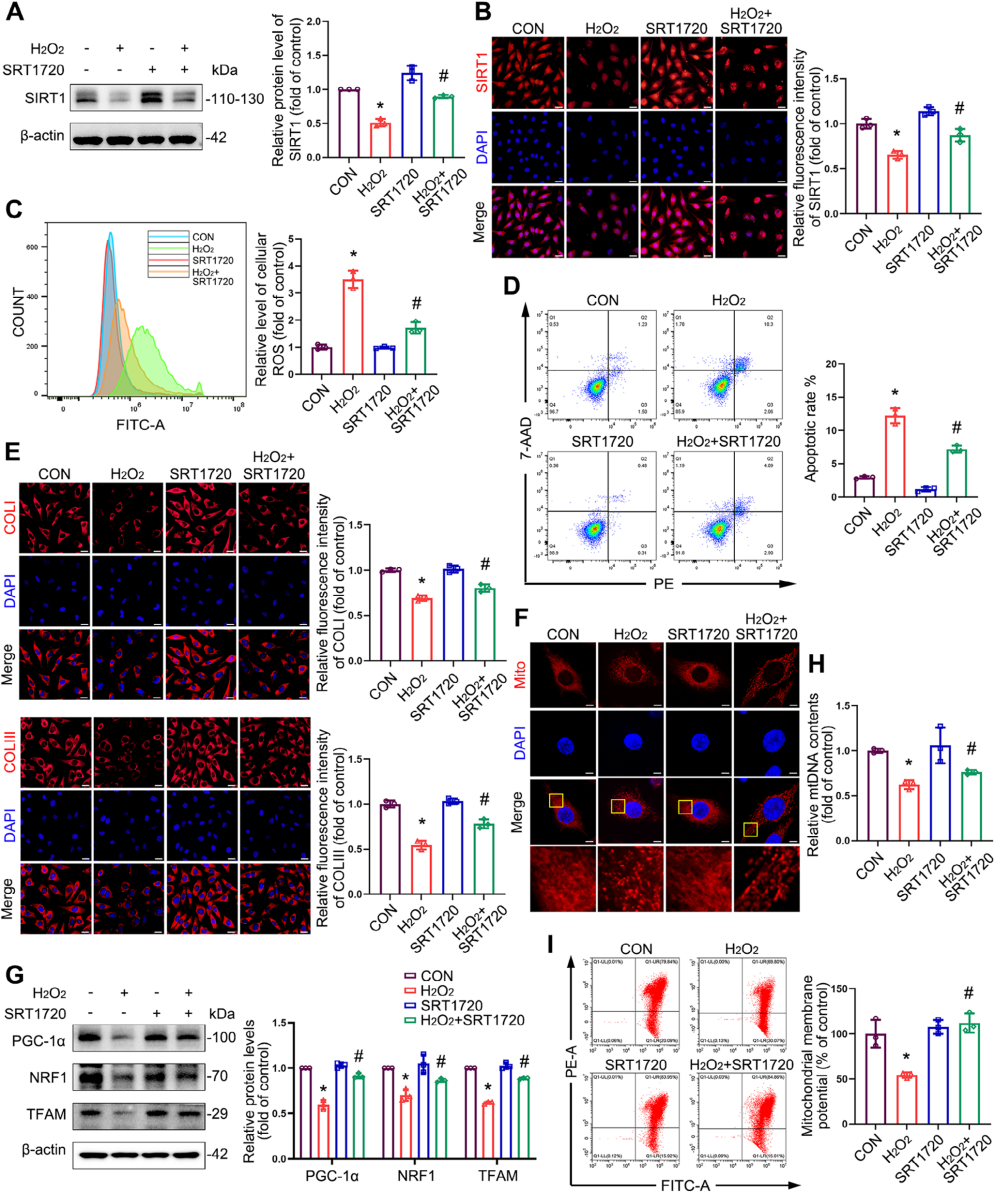
The effects of SIRT1 upregulation on mitochondrial morphology and mitochondrial biogenesis in L929 cells. L929 cells were exposed to 0.6 mM H_2_O_2_ for 4 h following a 12‐h treatment with the SIRT1 agonist SRT1720. (A) Immunoblot analysis of SIRT1 in L929 cells. Quantification represents the level of the indicated protein normalised to β‐Actin. (B) Immunofluorescence staining of SIRT1 in L929 cells. Scale bar = 20 μm. (C) Representative flow plots of ROS production in L929 cells measured by flow cytometry. (D) Flow cytometry analysis of apoptosis. (E) Immunofluorescence staining of collagen I (COL I) and collagen III (COL III) in L929 cells. Scale bar = 20 μm. (F) Representative images from MitoTracker‐stained control and H_2_O_2_‐treated L929 cells with or without SRT1720 treatment. Scale bars = 5 μm. (G) Immunoblot analyses of PGC‐1α, NRF1 and TFAM in L929 cells. Quantification represents the levels of the indicated proteins normalised to β‐Actin. (H) RT‐qPCR analyses of relative mtDNA contents in L929 cells. (I) Representative flow plots of MMP levels in L929 cells measured by flow cytometry. Data are expressed as mean ± SD. *p < 0.05 compared with the control group, ^#^ *p* < 0.05 compared with the H_2_O_2_‐treated group.

To investigate the role of SIRT1 in regulating mitochondrial function under oxidative stress, we used SRT1720, a SIRT1 agonist, in L929 cells with or without H₂O₂ (0.6 mM) treatment. Western blot and immunofluorescence analyses showed that SRT1720 significantly upregulated SIRT1 protein levels and circumvented the inhibitory effect of oxidative stress on SIRT1 expression (Figure 6A,B).

SRT1720 activation of SIRT1 mitigated intracellular ROS accumulation (Figure 6C), thereby relieving H₂O₂‐induced cellular apoptosis (Figure 6D) and increasing cellular viability (Figure S3A) with H₂O₂ exposure. In addition, we analysed the variation in collagen content and confirmed that the expression levels of collagens I and III were significantly augmented by SIRT1 activation under H₂O₂ treatment (Figure 6E).

Mitochondria stained using MitoTracker showed partially restored morphology from granular to linear tubular configuration following SIRT1 upregulation (Figure 6F), suggesting that SIRT1 activation improved mitochondrial abnormalities. We also determined whether mitochondrial biogenesis is improved by SIRT1 activation. Analysis of the expression of mitochondrial biogenesis‐related genes showed that SIRT1 activation increased mRNA and protein expression for members of the PGC‐1α/NRF1/TFAM axis (Figures S3B and 6G). Immunofluorescence of PGC‐1α, NRF1, and TFAM further supported the aforementioned observations (Figure S3C–E). SIRT1 activation also effectively increased mtDNA content (Figure 6H) and MMP (Figure 6I) under oxidative stress in L929 cells, rescuing mitochondrial biogenesis (Figure S3F).

**FIGURE 7:**
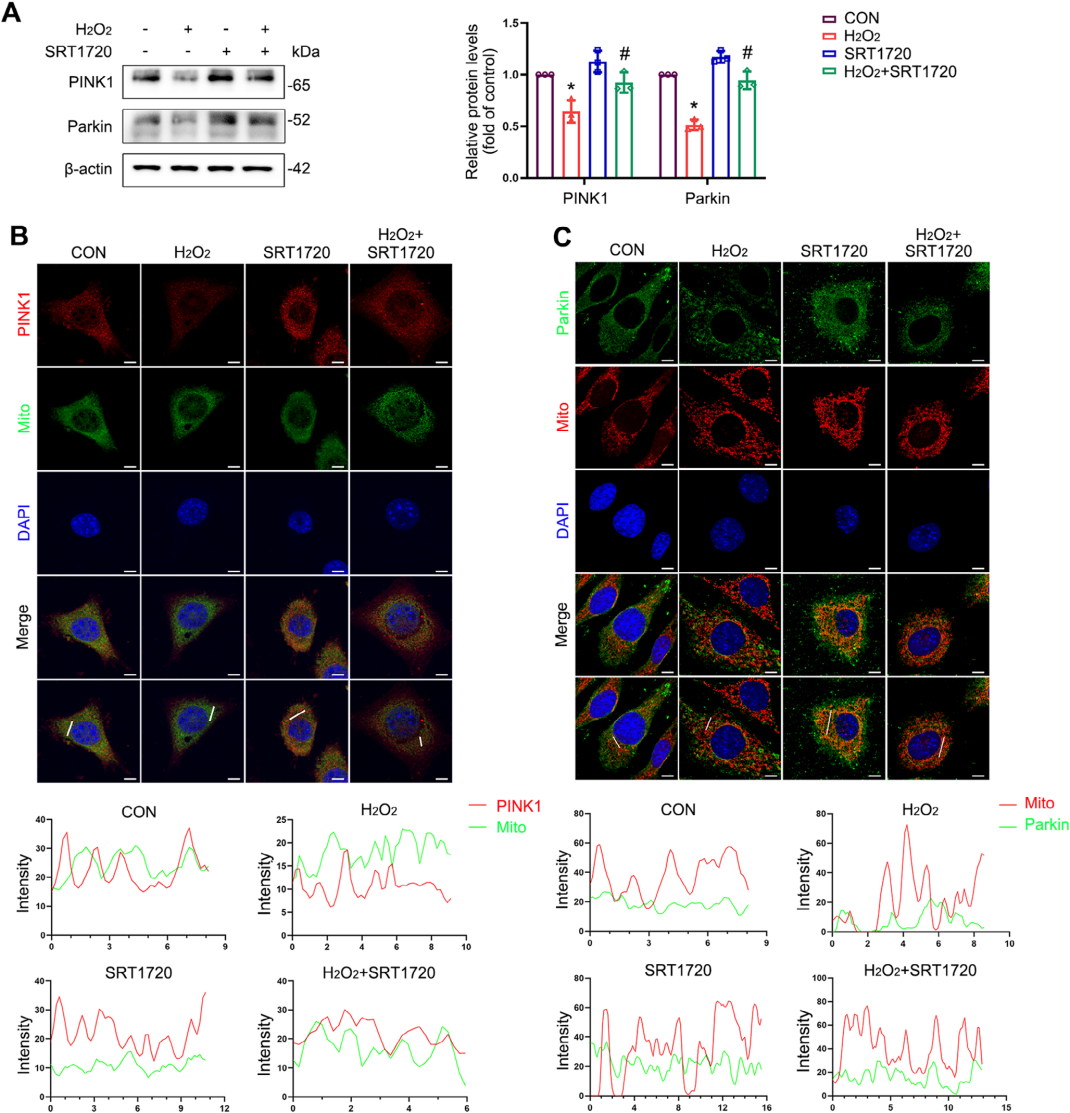
The effects of SIRT1 upregulation on mitophagy in L929 cells. (A) Immunoblot analyses of PINK1 and Parkin in L929 cells. Quantification represents the levels of the indicated proteins normalised to β‐Actin. (B, C) Co‐localization analysis of immunofluorescence images of PINK1 (red) and MitoTracker (green) (B), Parkin (green) and MitoTracker (red) (C) in different groups of L929 cells after being treated with H_2_O_2_ for 4 h with or without SRT1720. Data are expressed as mean ± SD. *p < 0.05 compared with control group, ^#^ *p* < 0.05 compared with H_2_O_2_ group.

To verify whether SIRT1 upregulation reversed the inhibition of mitophagy caused by oxidative stress, we examined PINK1 and Parkin protein levels in L929 cells with or without H_2_O_2_ or SRT1720 treatment. The results revealed that the expression of PINK1 and Parkin was elevated in SIRT1‐upregulated groups regardless of H_2_O_2_ exposure (Figures 7A and S3G). Immunofluorescence of the co‐localization of PINK1 and Parkin with the mitochondria also revealed that the increase in SIRT1 expression promoted the mitochondrial recruitment of PINK1 and Parkin in L929 cells under oxidative stress (Figure 7B,C).

These data indicate that SIRT1 upregulation alleviates mitochondrial morphological abnormalities and mitochondrial dysfunction induced by oxidative stress.

### 3.6 SIRT1 Upregulation Rescues the Structural Disruption of MAMs, Ameliorates ER Stress, and Restores Calcium Channel‐Related Proteins

**FIGURE 8:**
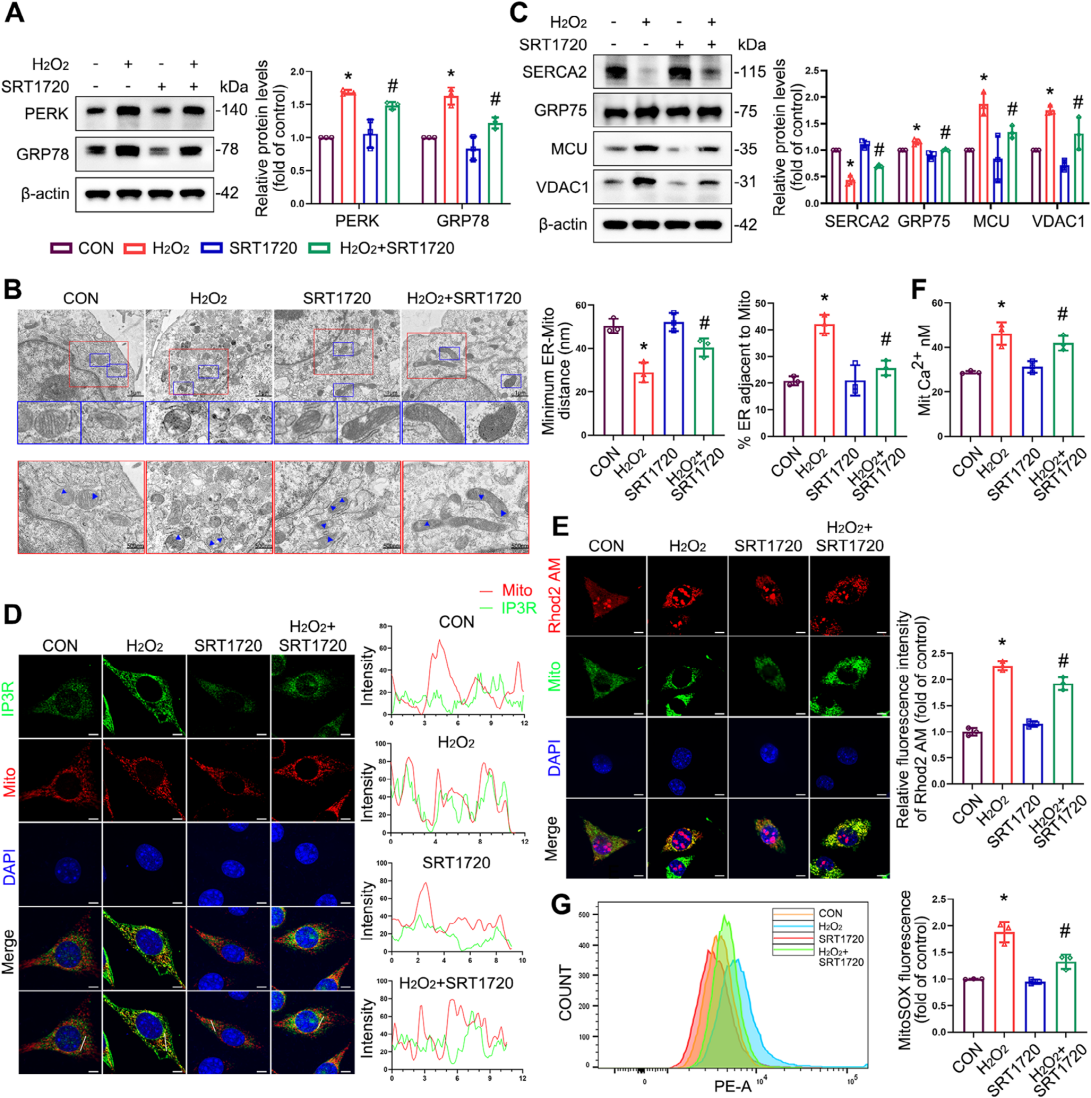
The effects of SIRT1 upregulation on structural and functional alterations of MAMs and calcium homeostasis in L929 cells under oxidative stress. (A) Immunoblot analyses of PERK and GRP78 in L929 cells. Quantification represents the levels of the indicated proteins normalised to β‐Actin. (B) Representative TEM images of mitochondrial ultrastructure of L929 cells. Blue arrows indicated MAMs. Quantitative analysis of MAM parameters, including MAM distance and length to mitochondrion in TEM images. (C) Immunoblot analyses of SERCA2, GRP75, MCU and VDAC1 in L929 cells. Quantification represents the levels of the indicated proteins normalised to β‐Actin. (D) Co‐localization analysis of immunofluorescence images of IP3R (green) and MitoTracker (red) in different groups of L929 cells. (E) Co‐localization fluorescence imaging was conducted with MitoTracker, demonstrating that Rhod‐2 AM is predominantly an indicator of mitochondrial Ca^2+^ levels. (F) Concentration of mitochondrial calcium ions. **(G)** Representative flow plots of mitochondrial ROS production in L929 cells measured by flow cytometry. Data are expressed as mean ± SD. *p < 0.05 compared with control group, ^#^ *p* < 0.05 compared with H_2_O_2_ group.

Treatment with SRT1720 alleviated H_2_O_2_‐induced ER stress (Figure 8A). TEM also showed that SIRT1 activation preserved the structural integrity of mitochondria, extended the distance between mitochondria and the ER, and reduced the length of MAMs (Figure 8B), suggesting that SIRT1 activation attenuated oxidative stress‐induced MAM formation. We further examined the impact of SIRT1 upregulation on calcium channel‐related proteins and found that the protein levels of VDAC1, MCU and GRP75 were significantly reduced following SRT1720 treatment, whereas those of SERCA2 increased remarkably (Figure 8C). Additionally, the co‐localisation of IP3R with VDAC1 and mitochondria weakened following SRT1720 treatment (Figures 8D and S3H). The structural restoration of MAMs by SIRT1 upregulation alleviated mitochondrial calcium overload (Figure 8E,F) and reduced mitochondrial ROS production (Figure 8G) in H_2_O_2_‐treated cells. These results suggest that SIRT1 upregulation rescues MAM disruption caused by oxidative stress and alleviates ER stress, thereby normalising calcium channel function and maintaining calcium homeostasis.

### 3.7 SIRT1 Upregulation Improves Urodynamics and Ameliorates Oxidative Damage Generated by VD in SUI Mice

**FIGURE 9:**
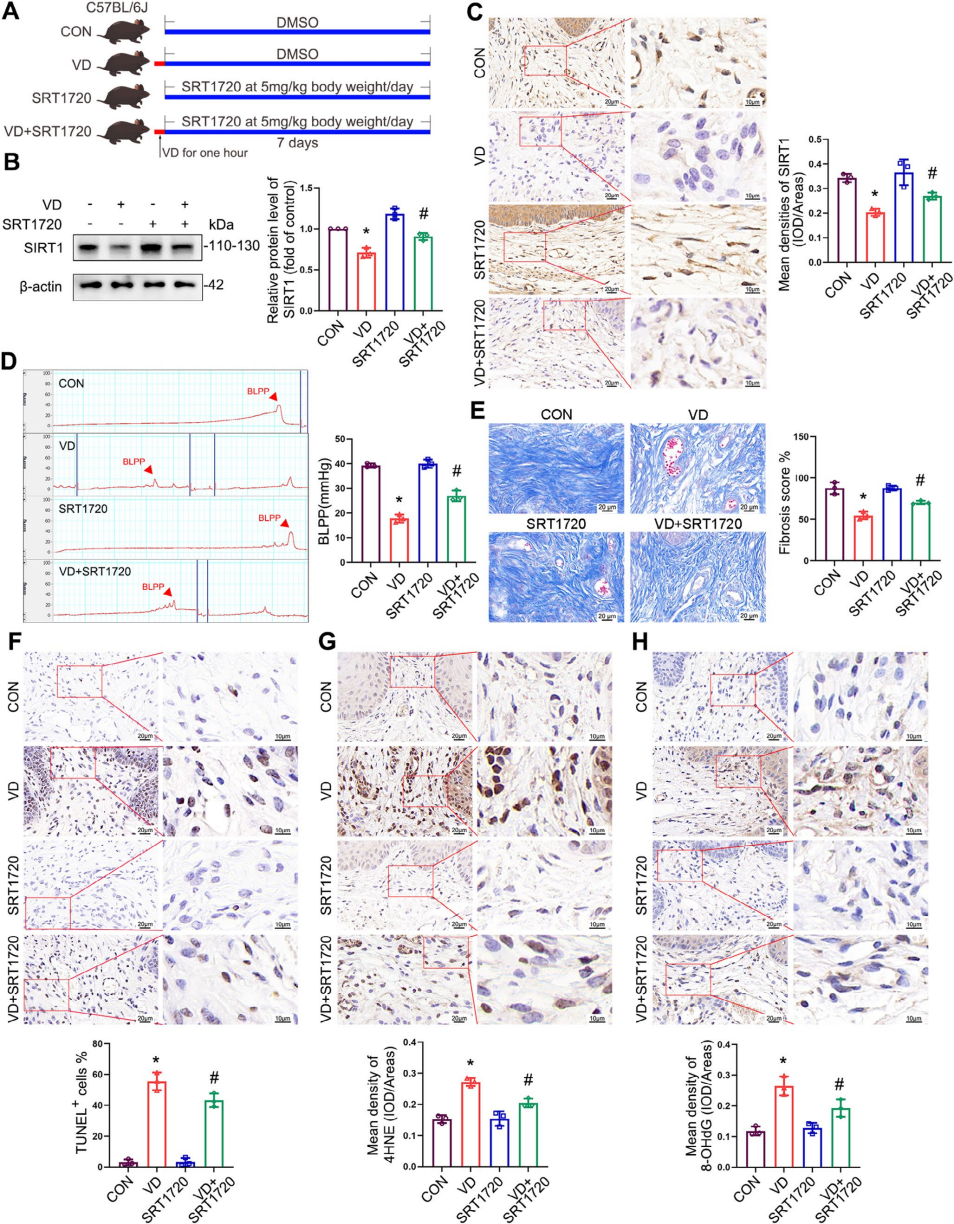
SIRT1 Upregulation improves urodynamics and alleviates oxidative damage caused by VD in SUI mice. (A) Experimental design of a cohort of offspring mice after VD treated with SRT1720 at different time points. (B) Immunoblot analysis of SIRT1 in the anterior vaginal wall tissues in mice. Quantification represents the level of the indicated protein normalised to β‐Actin. (C) Representative images of the IHC staining for SIRT1 in the anterior vaginal wall tissues were obtained and quantified. (D) The representativecurve of urinary dynamics of different groups, and statistical analysis of BLPP for different groups on 7 days. (E) Masson staining of collagen expression in the tissue of the anterior vaginal wall tissues in mice of four groups. (F) The detection of apoptosis in the anterior vaginal wall tissues of four groups was conducted using a TUNEL kit. (G, H) Representative images of the IHC staining of 4‐HNE (G) and 8‐OHdG (H) in the anterior vaginal wall tissues were obtained and quantified. Data are expressed as mean ± SD. *p < 0.05 compared with control group, ^#^ *p* < 0.05 compared with VD group.

To evaluate the therapeutic potential of SIRT1 in vivo, we established a mouse model of SUI via the VD, as previously described [30]. SRT1720 was injected into the mice at 5 mg/kg per day for 7 days (Figure 9A), and the mRNA and protein levels of SIRT1 were determined. The results showed that the expression of SIRT1 was significantly reduced in the anterior vaginal wall tissues of mice with SUI and could be upregulated by SRT1720 (Figures S4A and 9B). Immunohistochemical analysis also provided consistent results (Figure 9C). To confirm the therapeutic effects of SIRT1 in female mice with SUI, we measured the urodynamic parameter of bladder leak point pressure (BLPP) and assessed the histopathological changes in the anterior vaginal wall in the control and SUI mice with or without SRT1720. While our analysis showed that BLPP significantly declined in the SUI group compared with the control group, SIRT1 upregulation effectively circumvented the decrease in BLPP associated with VD (Figure 9D). Similarly, the decrease in collagen content and elevation in cellular apoptotic and oxidative damage markers precipitated by VD were significantly relieved by SRT1720 (Figure 9E–H). These results suggest that SIRT1 upregulation reduces the level of oxidative damage and increases collagen content in the anterior vaginal wall, thereby reflecting a therapeutic role in the SUI mouse model.

### 3.8 SIRT1 Upregulation Protects SUI Mice Against VD‐Induced Mitochondrial Dysfunction, Structural Disruption of MAMs, and Calcium Homeostasis Disruption

**FIGURE 10:**
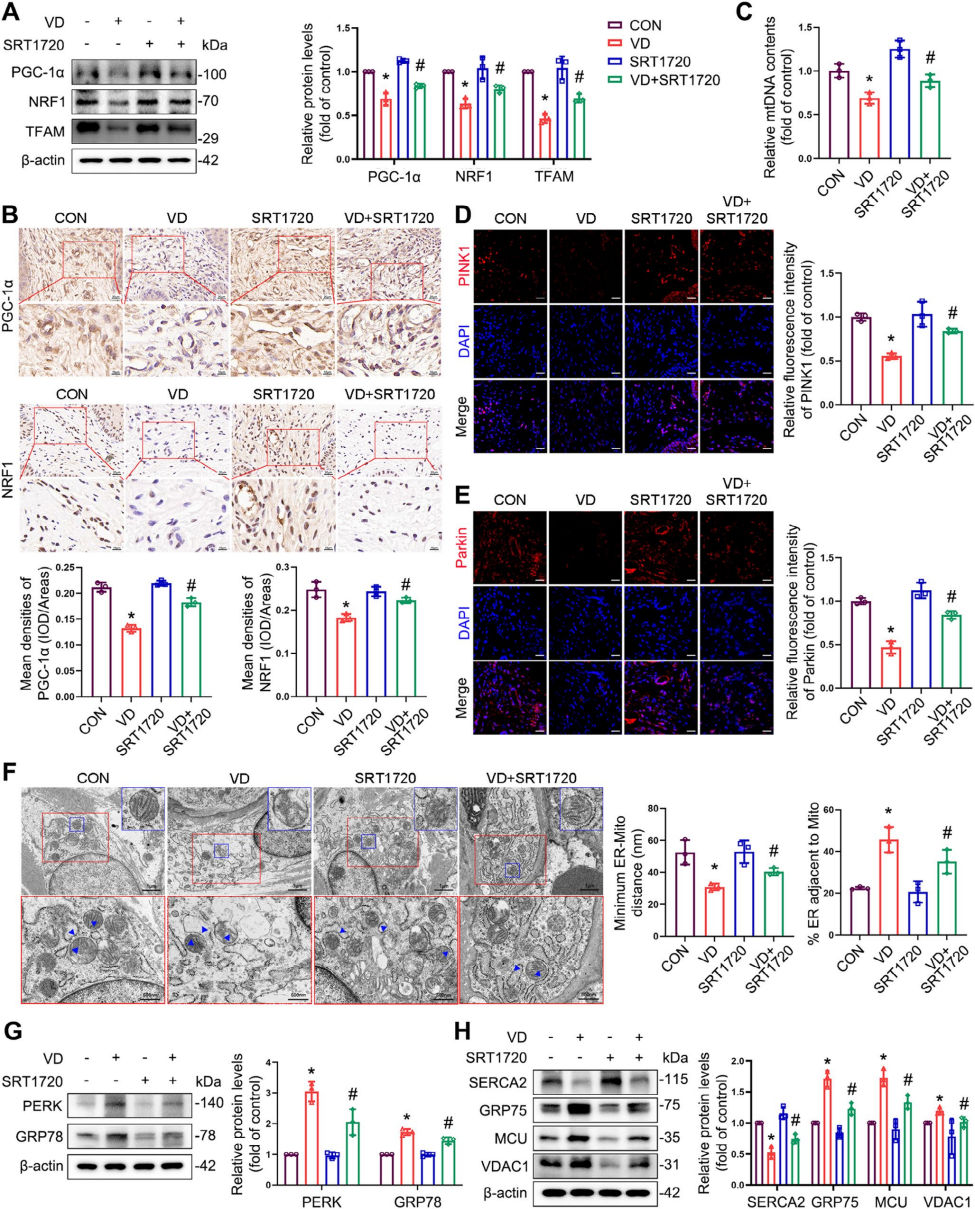
SIRT1 Upregulation improve mitochondrial function, MAM structural abnormalities, and calcium homeostasis in the anterior vaginal walls of SUI mice. (A) Immunoblot analyses of PGC‐1α, NRF1 and TFAM in the anterior vaginal wall tissues of mice. Quantification represents the levels of the indicated proteins normalised to β‐Actin. (B) Representative images of the IHC staining for PGC‐1α and NRF1 in the anterior vaginal wall tissues were obtained and quantified. (C) RT‐qPCR analyses of relative mtDNA contents in the anterior vaginal wall tissues of mice. (D, E) Immunofluorescence staining of PINK1 (D) and Parkin (E) in the anterior vaginal wall tissues of mice. Scale bar = 20 μm. (F) Representative TEM images of mitochondrial ultrastructure of fibroblasts in the anterior vaginal wall of mice. Blue arrows indicated MAMs. Quantitative analysis of MAM parameters, including MAM distance and length to mitochondrion in TEM images. (G) Immunoblot analyses of PERK and GRP78 in the anterior vaginal wall tissues of mice. Quantification represents the levels of the indicated proteins normalised to β‐Actin. (H) Immunoblot analyses of SERCA2, GRP75, MCU, and VDAC1 in the anterior vaginal wall tissues of mice. Quantification represents the levels of the indicated proteins normalised to β‐Actin. Data are expressed as mean ± SD. *p < 0.05 compared with control group, ^#^ *p* < 0.05 compared with VD group.

Based on our in vitro results, we investigated the effects of SIRT1 upregulation on mitochondrial biogenesis, mitophagy, and MAMs. The results demonstrated that the total mRNA and protein levels for PGC1α, NRF1, and TFAM in the anterior vaginal wall of mice with SRT1720 treatment were significantly augmented in SUI mice compared with the SUI group without SRT1720 treatment (Figures S4B and 10A). This was corroborated by the immunohistochemistry results (Figure 10B). In addition, RT‐qPCR revealed that the amount of mtDNA in the anterior vaginal wall of SUI mice was reduced compared with the control group, which was alleviated by SIRT1 upregulation (Figure 10C). Similarly, the relative expression levels of PINK1 and Parkin were increased in the anterior vaginal wall tissues of SRT1720‐treated SUI mice compared to those of SUI mice not treated with SRT1720 (Figure 10D,E). TEM of the anterior vaginal wall showed that mitochondria ruptured and vacuolated following VD in control mice, whereas mitochondrial damage was significantly reduced by SRT1720‐induced SIRT1 upregulation. When observing the structure of MAMs in the anterior vaginal wall of mice, we ascertained that the reduction in mitochondrial ER distance and the increase in MAM length caused by VD were significantly improved by SIRT1 activation (Figure 10F). SRT1720 treatment also alleviated ER stress and restored the expression levels of calcium channel‐related proteins in SUI mice (Figure 10G,H). Collectively, these results suggest that SRT1720 upregulates SIRT1, promotes mitochondrial biogenesis and mitophagy, rescues normal MAMs morphology and function, alleviates ER stress, restores cellular calcium homeostasis, and improves urodynamic results in SUI mice.

## 4 Discussion

SUI is a condition prevalent among middle‐aged and elderly women, impacting their quality of life by disrupting their physical, psychological, sexual, and social well‐being. Numerous theories on the pathophysiology of SUI have been proposed; yet, weaknesses in urethral supporting tissues, mainly the anterior vaginal wall, remain a major contributor to this condition [34, 35]. Recent studies have suggested that such weaknesses in urethral support are attributed to metabolic imbalances within the ECM in the anterior vaginal wall tissues [30, 36]. We have previously confirmed oxidative stress as an important pathological factor in ECM remodelling of the anterior vaginal wall and that antioxidant treatment significantly enhanced collagen expression in the anterior vaginal wall of SUI mice and improved urodynamic parameters [37]. Mitochondrial damage and dysfunction are the chief drivers of oxidative stress, promote each other, and lead to the onset and progression of multiple diseases [38, 39]. Diminished collagen content, increased cellular apoptosis, and elevated oxidative damage and ER stress in the present study were initially confirmed to be important pathological characteristics of the anterior vaginal wall tissues in patients with SUI. We also verified the therapeutic effects of SIRT1 on SUI in a cell model of oxidative stress and a mouse model of SUI, and speculated that the alleviation of mitochondrial dysfunction and dysregulation of mitochondria‐associated membranes comprised the mechanisms underlying these effects.

The intricate interplay between oxidative stress, ER stress, and mitochondria plays pivotal roles in maintaining cellular homeostasis, regulating metabolism, and responding to external stimuli [40, 41]. Thus, the delicate balance between ROS production and clearance is disrupted, resulting in progressive oxidative damage and, ultimately, cellular metabolic imbalance and apoptosis [42]. Multiple biological processes take place in the mitochondria, including mitochondrial biogenesis, maintenance of mtDNA homeostasis, and mitophagy, which are crucial for cellular metabolism and survival. Recent studies have shown that enhancing mitophagy and promoting mitochondrial biogenesis are the key regulators of mitochondrial quality control [43, 44]. The mitochondria were swollen, cristae were disrupted, and mitochondrial homeostasis was perturbed in the anterior vaginal wall of patients with SUI and SUI mice, consistent with the mitochondrial morphology in H_2_O_2_‐treated L929 cells. We also found that the mtDNA content was reduced in the anterior vaginal wall of patients with SUI and SUI mice, as well as in H_2_O_2_‐treated L929 cells, reflecting a decrease in mitochondrial numbers and inhibition of mitochondrial biogenesis. In addition, excessive ER stress was observed in the anterior vaginal wall of patients with SUI and SUI mice and in L929 cells subjected to oxidative stress injury. The ER is an effective modulator of mitochondrial function [45]. When ER stress persists, a substantial amount of Ca^2+^ is released from the ER and transferred to the mitochondria via MAMs, leading to mitochondrial calcium overload and dysfunction [46]. These dysfunctional mitochondria result in the excessive accumulation of intracellular ROS and further promote the release of Ca^2+^ from the ER to the mitochondria, causing ER Ca^2+^ depletion, sustained activation of ER stress, and oxidative stress exacerbation [47]. Using TEM, we observed in both in vivo *and* in vitro models that oxidative stress led to a shortened distance between the mitochondria and ER and increased the length of MAMs. Increased mitochondrial calcium overload, dysregulated calcium channel‐related proteins, and excess ROS production suggested a vicious cycle by the interplay between ER stress, mitochondrial dysfunction, and oxidative stress. Therefore, these may be important contributing factors to the looseness of the anterior vaginal walls observed in patients with SUI.

SIRT1 is important for regulating various cellular processes, such as metabolism, mitochondrial homeostasis, ER stress, and oxidative/anti‐oxidative balance [48, 49, 50]. Emerging evidence has suggested that SIRT1 is a potential therapeutic target in POP and operates via the inhibition of oxidative stress and cellular senescence in human uterosacral ligament fibroblasts [27]. Our data verified a reduction in SIRT1 expression in the anterior vaginal wall of patients with SUI and SUI mice, as well as in H_2_O_2_‐treated L929 cells, indicating that oxidative stress inhibited the expression of SIRT1. PGC‐1α is a powerful transcriptional co‐regulator of mitochondrial genes, interacts with NRF1 and TFAM, and modulates multiple biological processes within mitochondrial metabolism, including mitochondrial biogenesis and mitochondrial oxidative stress [51, 52]. In our study, the PGC‐1α/NRF1/TFAM‐signalling pathway was significantly repressed by oxidative stress, both in vivo and in vitro. Mitophagy is a fundamental process in mitochondrial quality control that selectively removes excess or damaged mitochondria via autophagy through the PINK1/Parkin pathway. Previous studies have shown that this pathway is inhibited by oxidative stress induced by ROS overgeneration [53], consistent with our results. The activation of mitophagy leads to PINK1 phosphorylation in Parkin, promoting its translocation to the mitochondria. Under the action of LC3, Parkin aggregates into bilayered autophagic vesicles and is ultimately degraded in lysosomes [54]. Therefore, we examined the co‐localisation of PINK1 and Parkin with mitochondria following H_2_O_2_ treatment. The results showed that oxidative stress reduced the recruitment of both molecules to mitochondria, prevented mitophagy, and reduced PINK1/Parkin expression in the vaginal walls of women with SUI and a mouse model of SUI. We, thus, hypothesised that SIRT1 suppression inhibited the PGC‐1α/NRF1/TFAM‐ and the PINK1/Parkin‐signalling pathways under oxidative stress.

We used SRT1720, a potent SIRT1 agonist [55], to overexpress SIRT1 and observed a reduction in mitochondrial and total ROS and enhancement of MMP in H_2_O_2_‐treated L929 cells. In addition, our data revealed augmented expression of PGC‐1α/NRF1/TFAM‐axis members and elevated mtDNA following SIRT1 upregulation, suggesting that SIRT1 activation promotes mitochondrial biogenesis. To confirm whether SIRT1 upregulation exerted an effect on mitophagy, we explored PINK1 and Parkin expression following SRT1720 treatment and demonstrated that SIRT1 activation increased the expression of both proteins, recruited additional PINK1 and Parkin to the mitochondria, and activated mitophagy to remove damaged mitochondria. Our In vivo data further validated SIRT1 upregulation as promoting mitochondrial biogenesis and mitophagy and improving mitochondrial morphological damage by activating the PGC‐1α/NRF1/TFAM and PINK1/Parkin axes. These actions effectively improved urodynamic parameters in the SUI mouse model. Although oxidative stress is exacerbated by MAM damage and mitochondrial ER calcium dysregulation, whether SIRT1 activation mitigates oxidative damage is yet to be determined. Herein, SIRT1 activation alleviated ER stress and regulated the expression of calcium channel‐related proteins. Moreover, SIRT1 activation increased the distance between the mitochondria and ER while shortening the length of MAMs both in vivo and in vitro. These reduced Ca^2+^ flux from the ER to the mitochondria and promoted ER calcium uptake. We confirmed that SIRT1 upregulation reversed the inhibition of the PGC‐1α/NRF1/TFAM and the PINK1/Parkin‐signalling pathways following oxidative damage; this allowed for the promotion of mitochondrial biogenesis and mitophagy. Moreover, SIRT1 upregulation alleviated the vicious cycle of interplay among ER stress, mitochondrial dysfunction, and oxidative stress by improving the structure of MAMs and calcium homeostasis, ultimately restoring mitochondrial function in fibroblasts and the repair of anterior vaginal walls in women with SUI.

In conclusion, this study uncovered a novel pathological mechanism and a potential therapeutic target for SUI. The therapeutic efficacy of SIRT1 in women with SUI is associated with the amelioration of mitochondrial biogenesis and mitophagy. Moreover, SIRT1 alleviates mitochondrial calcium overload, ER and oxidative stress by ameliorating the structure of MAMs and regulating calcium channels. Our findings provide novel insights into the pathogenesis of SUI and a theoretical basis for future studies on SUI. However, this study has some limitations. First, concerning SIRT1 intervention, the construction of gene‐overexpressing cells and gene‐knockout mice provided more convincing evidence than agonist experiments alone. Second, the specific mechanism underlying the inhibition of SIRT1 in the anterior vaginal wall in patients with SUI remains unknown. In subsequent studies, we intend to further investigate the specific role of SIRT1 and the associated mechanism of action in SUI pathogenesis using SIRT1‐gene intervention in cellular and mouse models.

## Author Contributions

**Liying Chen:** writing – original draft, visualisation, data curation, investigation, formal analysis, validation, methodology, conceptualisation. **Jianming Tang:** writing – original draft, data curation, resources, methodology, conceptualisation, funding acquisition. **Xiaohu Zuo:** methodology, visualisation, investigation, writing – original draft. **Bingshu Li:** investigation, formal analysis, writing – original draft. **Cheng Liu:** investigation, formal analysis, writing – original draft. **Shasha Hong:** investigation, formal analysis, writing – original draft. **Jie Min:** investigation, formal analysis, writing – original draft. **Ming Hu:** investigation, formal analysis, writing – original draft. **Suting Li:** visualisation, writing – original draft. **Min Zhou:** funding acquisition, writing – review and editing. **Mao Chen:** validation, methodology, writing – original draft. **Yong He:** validation, methodology, writing – original draft. **Ya Xiao:** investigation, methodology, writing – original draft. **Xiaoyu Huang:** investigation, methodology, writing – original draft. **Li Hong:** funding acquisition, project administration, supervision, writing – review and editing, resources, validation, conceptualisation.

## Conflicts of Interest

The authors declare no conflicts of interest.

## Supporting information


**Data S1.** Supporting Information.

## Data Availability

The data supporting the findings of this study are available from the corresponding author upon reasonable request.
