# Supplementary material for: SIRT1 Alleviates Oxidative Stress‐Induced Mitochondrial Dysfunction and Mitochondria‐Associated Membrane Dysregulation in Stress Urinary Incontinence
Source: Cell Prolif. 2025 Feb 21;58(5):e70009. doi: 10.1111/cpr.70009 (PMC12099215; doi:10.1111/cpr.70009)
Supplement: Supplementary file 1 — Data S1. Supporting Information. [file CPR-58-e70009-s001.docx]

**SIRT1 Alleviates Oxidative Stress-induced Mitochondrial Dysfunction and Mitochondria-associated Membrane Dysregulation in Stress Urinary Incontinence**

***Supplementary materials***

**Supplementary** **tables**

**Table S1. Sequences of primers used in RT-qPCR for mRNA detections**

| Gene | Species | Forward primer | Reverse primer |
| --- | --- | --- | --- |
| SIRT1 | HUMAN | TTCCAAGTTCCATACC | TTCACCACCTAACCTATGACACAATTC |
| PGC-1α | HUMAN | GACACAACACGGACAGAACTGAG | GCATCACAGGTATAACGGTAGGTAATG |
| NRF1 | HUMAN | ATAGTATGCTGAGTGCTGATGAAGAC | GCTGCTGTGGAGTTGAGTATATCTG |
| TFAM | HUMAN | TTCATCTGTCTTGGCAAGTTGTCC | TGGGTTCTGAGCTTTAAATATGGGTAG |
| GAPDH | HUMAN | AGAAGGCTGGGGCTCATTTG | AGGGGCCATCCACAGTCTTC |
| SIRT1 | MICE | CTCCACGAACAGCTTCACAATCAAC | CCAGACCTCCCAGACCCTCAAG |
| PGC-1α | MICE | CTCCCGCTTCTCGTGCTCTTTG | ATGTGTCGCCTTCTTGCTCTTCC |
| NRF1 | MICE | TGAATTACTCTGCTGTGGCTGATGG | CCTCTGATGCTTGCGTCGTCTG |
| TFAM | MICE | TCGATTTTCCACAGAACAGCTACCC | TTTCCCTGAGCCGAATCATCCTTTG |
| GAPDH | MICE | TCACCATCTTCCAGGAGCGAGAC | TGAGCCCTTCCACAATGCCAAAG |

**Table S2. Sequences of primers used in RT-qPCR for measurement of relative mtDNA contents**

| Gene | Species | Forward primer | Reverse primer |
| --- | --- | --- | --- |
| mtND4 | HUMAN | CTCATCAGTAAGCCATATAGC | TTCGTTCGTAGTTGGTGTT |
| mtGAPDH | HUMAN | GGAAGGACTCATGACCACAGT | GCCATCACGCCACAGTTTC |
| mtAtp6 | MICE | GCCATTCCACTATGAGCTGGAGCC | GTGGAAGGAAGTGGGCAAGTGAGC |
| Tert | MICE | CTAGCTCATGTGTCAAGACCCTCTT | GCCAGCACGTTTCTCTCGTT |

**Supplementary** **figures**


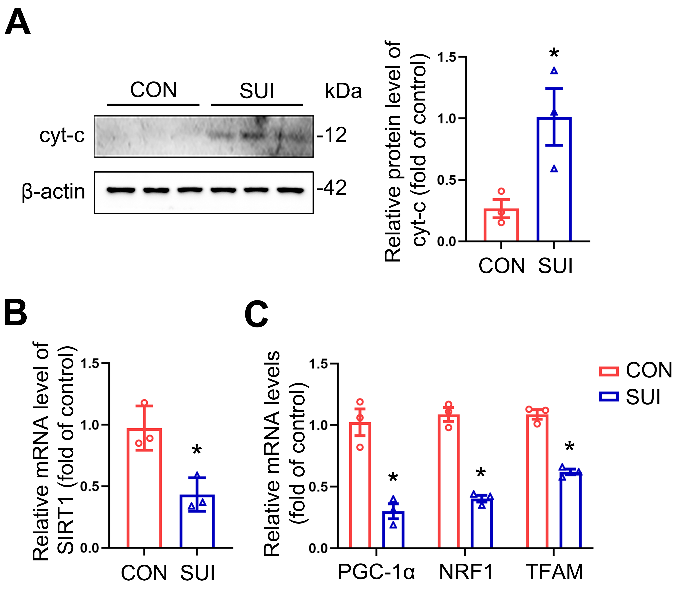


**Figure S1. Pathological differences of the anterior vaginal wall in patients with SUI. (A)** Immunoblot analyses of Cytochrome c in human anterior vaginal wall. Quantification represents the level of the indicated protein normalized to β-actin. **(B)** RT-qPCR analysis of SIRT1 mRNA content in the anterior vaginal wall of SUI patients. **(C)** RT-qPCR analyses of PGC-1α, NRF1 and TFAM mRNA contents in the anterior vaginal wall of SUI patients. Data are expressed as mean ± SD. **P* < 0.05 compared with control group.


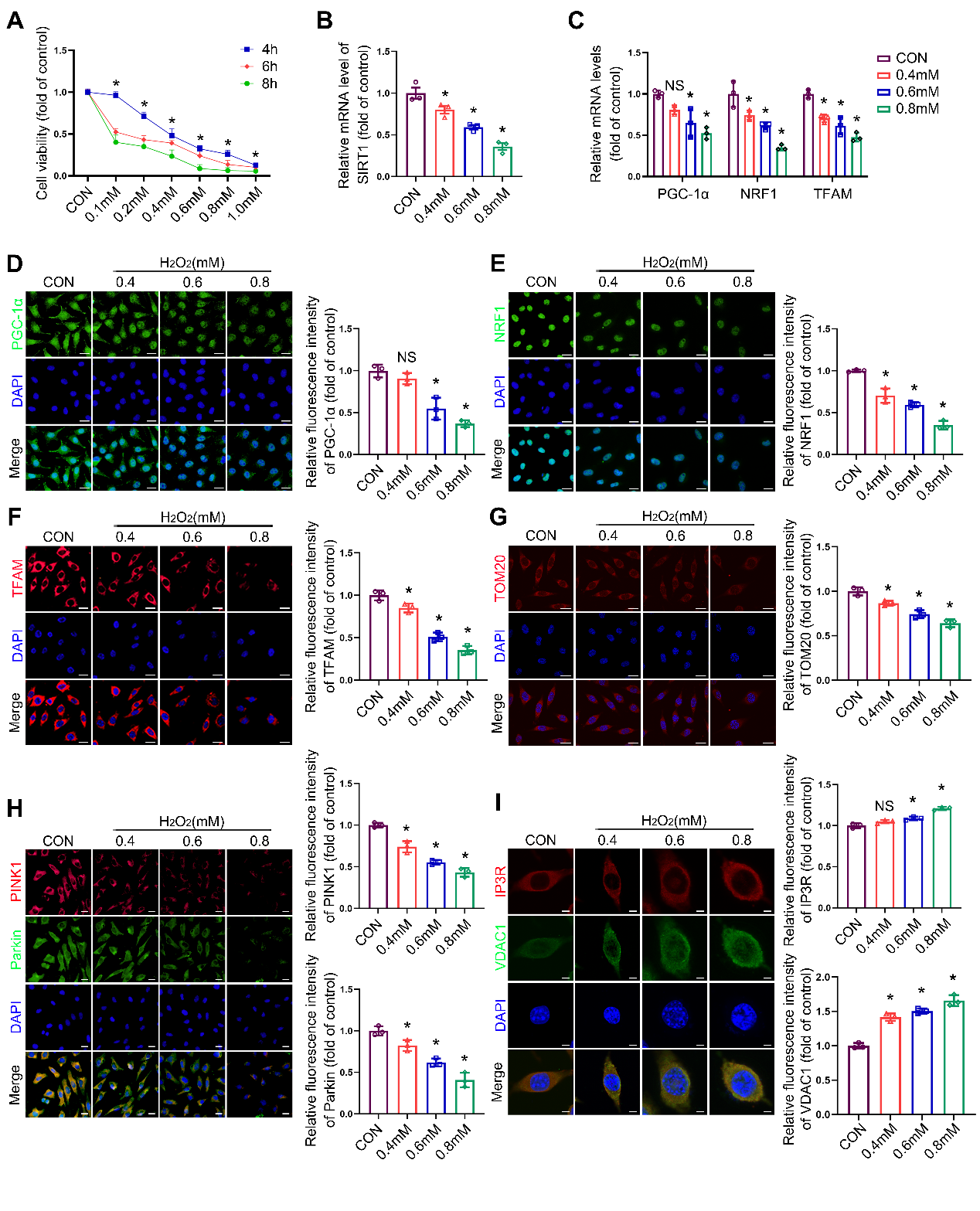


**Figure S2.** **Oxidative stress inhibits cell proliferation and induces mitochondrial dysfunction. (A)** The viability level of L929 cells was determined by CCK-8 assay. L929 cells were treated with 0.4 mM, 0.6 mM, and 0.8 mM H_2_O_2_ for 4, 6, and 8 hours. **(B)** RT-qPCR analysis of SIRT1 mRNA content in L929 cells. **(C)** RT-qPCR analyses of PGC-1α, NRF1, and TFAM mRNA contents in L929 cells. **(D-F)** Immunofluorescence staining of PGC-1α **(D),** NRF1 **(E),** and TFAM **(F)** in L929 cells. Scale bar = 20 μm. **(G)** Immunofluorescence staining of Tom20 in L929 cells. Scale bar = 20 μm. **(H)** Immunofluorescence staining of PINK1 and Parkin in L929 cells. Scale bar = 20 μm. **(I)** Immunofluorescence staining of IP3R and VDAC1 in L929 cells. Scale bar = 20 μm. Data are expressed as mean ± SD. **P* < 0.05 compared with control group. NS: no significant difference was observed.


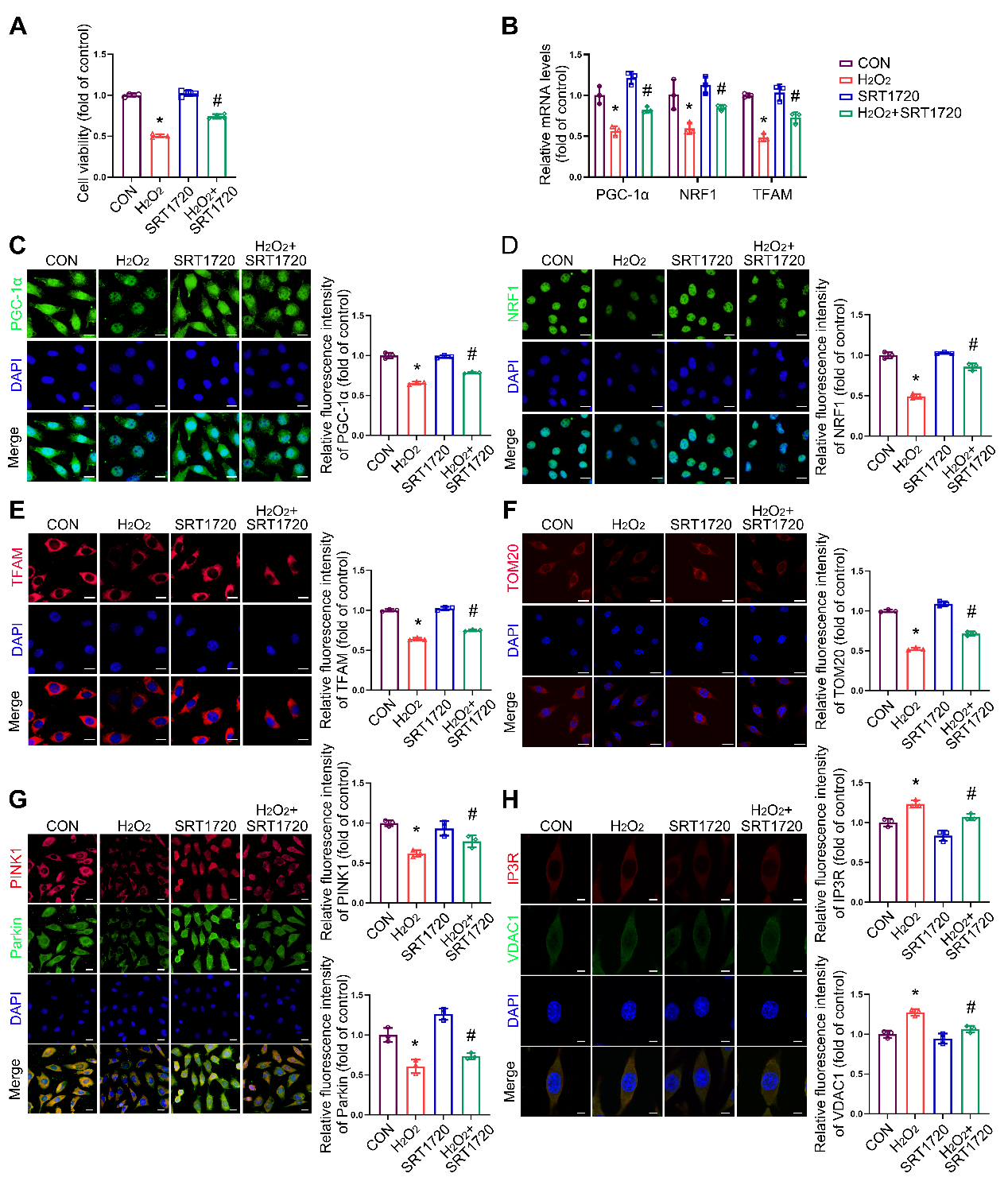


**Figure S3.** **SIRT1-upregulation alleviated oxidative stress induced inhibition of cell proliferation and induced mitochondrial dysfunction. (A)** The viability level of L929 cells was determined by CCK-8 assay. L929 cells were treated with 0.6 mM H_2_O_2_ for 4 hours after SRT1720 treatment. **(B)** RT-qPCR analysis of PGC-1α, NRF1, and TFAM mRNA contents in L929 cells. **(C-E)** Immunofluorescence staining of PGC-1α **(C),** NRF1 **(D),** and TFAM **(E)** in L929 cells. Scale bar = 20 μm. **(F)** Immunofluorescence staining of Tom20 in L929 cells. Scale bar = 20 μm. **(G)** Immunofluorescence staining of PINK1 and Parkin in L929 cells. Scale bar = 20 μm. **(H)** Immunofluorescence staining of IP3R and VDAC1 in L929 cells. Scale bar = 20 μm. Data are expressed as mean ± SD. **P* < 0.05 compared with control group, #*P* < 0.05 compared with H_2_O_2_ group.

**
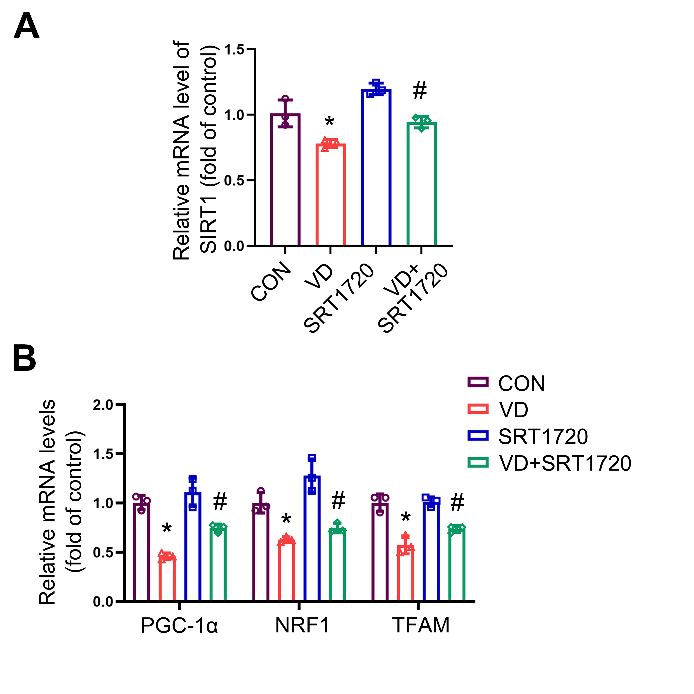
**

**Figure S4.** **mRNA expression levels of mitochondrial biogenesis-related genes in mice. (A)** RT-qPCR analysis of SIRT1 mRNA content in the anterior vaginal wall of mice. **(B)** RT-qPCR analyses of PGC-1α, NRF1, and TFAM mRNA contents in the anterior vaginal wall of mice. Data are expressed as mean ± SD. **P* < 0.05 compared with control group, #*P* < 0.05 compared with VD group.
